# Interaction of Full-Length Glycosylphosphatidylinositol-Anchored Proteins with Serum Proteins and Their Translocation to Cells In Vitro Depend on the (Pre-)Diabetic State in Rats and Humans

**DOI:** 10.3390/biomedicines9030277

**Published:** 2021-03-10

**Authors:** Günter A. Müller, Andreas Lechner, Matthias H. Tschöp, Timo D. Müller

**Affiliations:** 1 Institute for Diabetes and Obesity (IDO), Helmholtz Diabetes Center (HDC) at Helmholtz Zentrum München, German Research Center for Environmental Health (GmbH), Ingolstädter Landstraße 1, 85764 Oberschleissheim, Germany; timo.mueller@helmholtz-muenchen.de; 2 German Center for Diabetes Research (DZD), 85764 Oberschleissheim, Germany; matthias.tschoep@helmholtz-muenchen.de; 3 Department Biology I, Genetics, Ludwig-Maximilians-Universität München, 82152 Planegg-Martinsried, Germany; 4 Diabetes Reseach Group, Medical Center, Medizinische Klinik IV, Ludwig-Maximilians-Universität München, 80336 München, Germany; andreas.lechner@med.uni-muenchen.de; 5 Clinical Cooperation Group Type 2 Diabetes, Helmholtz Zentrum München, 85764 Oberschleissheim, Germany; 6 Division of Metabolic Diseases, Department of Medicine, Technische Universität München, 81675 München, Germany; 7 Helmholtz Zentrum München, German Research Center for Environmental Health (GmbH), 85764 Oberschleissheim, Germany; 8 Department of Pharmacology and Experimental Therapy, Institute of Experimental and Clinical Pharmacology and Toxicology, Eberhard Karls University Hospitals and Clinics, 72076 Tübingen, Germany

**Keywords:** glycosylphosphatidylinositol (GPI)-anchored proteins (GPI-APs), GPI-specific phospholipase D (GPLD1), insulin resistance, prediction of metabolic diseases

## Abstract

Glycosylphosphatidylinositol (GPI)-anchored proteins (GPI-APs), which are anchored at the surface of mammalian cultured and tissue cells through a carboxy-terminal GPI glycolipid, are susceptible to release into incubation medium and (rat and human) blood, respectively, in response to metabolic stress and ageing. Those GPI-APs with the complete GPI still attached form micelle-like complexes together with (lyso)phospholipids and cholesterol and are prone to degradation by serum GPI-specific phospholipase D (GPLD1), as well as translocation to the surface of acceptor cells in vitro. In this study, the interaction of GPI-APs with GPLD1 or other serum proteins derived from metabolically deranged rat and humans and their translocation were measured by microfluidic chip- and surface acoustic wave-based sensing of micelle-like complexes reconstituted with model GPI-APs. The effect of GPI-AP translocation on the integrity of the acceptor cell surface was studied as lactate dehydrogenase release. For both rats and humans, the dependence of serum GPLD1 activity on the hyperglycemic/hyperinsulinemic state was found to be primarily based on upregulation of the interaction of GPLD1 with micelle-like GPI-AP complexes, rather than on its amount. In addition to GPLD1, other serum proteins were found to interact with the GPI phosphoinositolglycan of full-length GPI-APs. Upon incubation of rat adipocytes with full-length GPI-APs, their translocation from the micelle-like complexes (and also with lower efficacy from reconstituted high-density lipoproteins and liposomes) to acceptor cells was observed, accompanied by upregulation of their lysis. Both GPI-AP translocation and adipocyte lysis became reduced in the presence of serum proteins, including (inhibited) GPLD1. The reduction was higher with serum from hyperglycemic/hyperinsulinemic rats and diabetic humans compared to healthy ones. These findings suggest that the deleterious effects of full-length GPI-APs following spontaneous release into the circulation of metabolically deranged rats and humans are counterbalanced by upregulated interaction of their GPI anchor with GPLD1 and other serum proteins. Thereby, translocation of GPI-APs to blood and tissue cells and their lysis are prevented. The identification of GPI-APs and serum proteins interacting within micelle-like complexes may facilitate the prediction and stratification of diseases that are associated with impaired cell-surface anchorage of GPI-APs, such as obesity and diabetes.

## 1. Introduction

About 2% of the cell-surface proteins in mammals are anchored at the outer leaflet of the phospholipid bilayer of plasma membranes through a glycosylphosphatidylinositol (GPI) moiety [1,2,3]. This glycolipid is covalently coupled via a phosphoethanolamine bridge to the carboxy-terminus of a typically large hydrophilic protein moiety [4,5,6]. Since the first identification of GPI-anchored proteins (GPI-APs) [7,8], it has been speculated that this type of membrane anchorage could favor their spontaneous nonenzymic release from plasma membranes (see [9,10] for reviews). This has to be discriminated from the enzymic release, which has been amply documented in the past [11,12]. The possibility of spontaneous release was supported by the considerably lower forces of binding to and extraction from supported phospholipid/cholesterol mono- and bilayers for GPI-APs compared to transmembrane proteins as measured in various biophysical studies [13,14,15,16,17].

Until recently, the experimental evidence for the (patho)physiological relevance of the spontaneous release of GPI-APs has remained limited. GPI-APs that had retained the complete GPI moiety were identified in multimeric high-molecular aggregates in body fluids, such as seminal plasma [18]; microvesicles and exosomes released from blood and tissue cells [19,20,21,22,23]; lipoprotein-like particules, such as milk fat globules [24] and intestinal surfactant-like particles [25]; and lipoproteins [26,27]. Recently, full-length GPI-APs were detected in the incubation medium of isolated rat adipocyte plasma membranes and cultured rat adipocytes in vitro, as well as in serum of metabolically deranged rats and humans [28] or old rats [29]. Importantly, they were found to be embedded together with (lyso)phospholipids and cholesterol in micelle-like complexes, called micelle-like GPI-AP complexes, rather than in lipid-free aggregates, vesicles, or lipoproteins [30].

More than four decades ago, the GPI-AP acetylcholinesterase (AChE) was reported to be transferred from intact human erythrocytes to protein-free sealed liposomes upon incubation [31]. The possibility of transfer was subsequently extended to other GPI-APs and “empty” phospholipid bi- and monolayers [32,33,34,35]. Together, these findings raised the possibility for intercellular transfer of full-length GPI-APs as a consequence of their release from the surface of mammalian “donor” cells and subsequent translocation to “acceptor” cells at the same or distant tissue depot(s). Therefore, it is tempting to speculate that the concentration of full-length GPI-APs in extracellular fluids has to be kept low in order to avoid unspecific and deleterious surface expression of GPI-APs at cells which do not produce them.

One protective mechanism may rely on the cleavage of the GPI anchor by GPI-specific phospholipase D (GPI-PLD) activity, which in mammalian serum is constituted solely by GPI-PLD1 (GPLD1), according to our current knowledge [36,37,38]. Recent studies revealed that the serum concentration of full-length GPI-APs is correlated to serum GPLD1 activity and blood glucose/plasma insulin levels in diabetic rats and human patients [28,30]. This was interpreted as counterregulation between the release of full-length GPI-APs into the circulation due to metabolic stress and their lipolytic degradation.

The present study demonstrates dependencies between the metabolic state of rats, as well as humans, and (i) the interaction of GPLD1 and other serum proteins with micelle-like GPI-AP complexes, (ii) the translocation of GPI-APs from micelle-like complexes to rat adipocytes, and (iii) the sensitivity of rat adipocytes toward lysis upon exposure to GPI-APs and serum proteins. The corresponding assays were based on a recently developed homogenous and microfluidic-chip-based sensor system. This relies on the propagation of horizontal surface acoustic waves (SAWs) along a gold chip surface. It is affected by binding of any entities to the chip either directly or via specific capturing molecules or interaction partners of interest immobilized to the chip with the aid of standard coupling procedures. The resulting amplitude reductions and right-ward phase shifts of the SAW represent measures for the increased viscosity (i.e., alterations in the biophysical properties) and loaded mass (i.e., presence and amount) of the captured and interacting entities as the sample analytes. Specific capture of GPI-APs by the chip surface is guaranteed by covalent coupling to the chip of α-toxin, which binds to the core glycan of GPI-APs with high specificity [39,40]. The demonstration of (lyso)phospholipids in complex with the GPI-APs is then performed by binding “in sandwich” of the phosphatidylserine-binding protein annexin-V and appropriate antibodies to the captured GPI-APs. Similarly, the presence of serum proteins interacting with GPI-APs is monitored as SAW phase shift upon sample injection into the chip. The major advantages of SAW sensing compared to optical laser-based methods are its compatibility with turbid and complex analytes and matrices (e.g., lipids, membranes), as well as its exquisite sensitivity ([28,41,42]; see also the Supplementary Materials herein for further theoretical and experimental details and references).

The findings suggest that the interaction of serum proteins, among them GPLD1, with the GPI anchor of GPI-APs, which are released into the circulation in response to metabolic derangement, inhibits their translocation from micelle-like complexes to cell surfaces. This may prevent “acceptor” cells from deleterious consequences provoked by GPI-APs after their “unwanted” transfer from “donor” cells.

## 2. Materials and Methods

### 2.1. Materials

Male Wistar rats (Crl:WI(WU)) were obtained from Charles River (Sulzfeld, Germany). See Supplementary Materials.

### 2.2. Animal Handling

Rats were housed two per cage in an environmentally controlled room with a 12:12-h light–dark circle (light on at 06:00) and ad libitum access to water and standard rat chow (17.7 kJ/g, Ssniff diet R/M-H, V1535 with 18% crude protein, 4.7% sugar, and 3.5% crude fat) (Ssniff, Soest, Germany). See Supplementary Materials.

### 2.3. GPLD1 Activity Assay Using Micelle-Like Bovine Erythrocyte AChE (bAChE) Complexes and Chip-Based Sensing, Preparation, and Enrichment of hCD73 from Human Erythrocytes, Measurement of Cholesterol and HDL Concentrations in Human Plasma, and Reconstitution of Micelle-Like Complexes without bAChE

See Supplementary Materials.

### 2.4. Determination of the Amount of GPLD1 Protein in Serum Using Chip-Based Sensing

For the generation of chips that capture GPLD1, protein A (50 mg/mL in PBS, 0.1 mM EDTA, 10% glycerol) diluted 10-fold in immobilization buffer (10 mM sodium acetate, pH 5.5) was coupled to the channels of activated long-chain 3D carboxymethyl (CM) dextran chips (SAW Instruments Inc., Bonn, Germany) in a SamX instrument (SAW Instruments Inc.) as described previously [28], with the following modifications: A 200-μL portion of protein A was injected at a flow rate of 40 µL/min. After washing with 300 µL of running buffer PBST (PBS containing 0.005% Tween 20 [*v*/*v*]) at a flow rate of 150 µL/min, the residual activated groups on the chip surface were capped by injecting 240 µL of 1 M ethanolamine (pH 8.5) at a flow rate of 80 µL/min. For the generation of a “blank” channel, one channel was activated and capped upon injection of buffer (instead of protein A). Following washing with 300 µL of PBST at a flow rate of 150 µL/min, 100 µL of anti-GPLD1 antibody, diluted 20-fold with running buffer, were injected at a flow rate of 15 µL/min. After washing with 300 µL of PBST at a flow rate of 150 µL/min, 100 (1×), 200 (2×) or 500 (5×) µL portions of serum, diluted 20-fold with PBST, were injected at a flow rate of 20 (1×), 40 (1×) and 100 (5×) µL/min. Measurement of the phase shift was performed at 22 °C. Start and termination points of the sample injections or washing cycles are indicated with green and black arrows, respectively, in the figures. Chips were regenerated by successive injections of 60 µL of 10 mM glycine (pH 3.5) and 30 µL of 4 M urea with waiting for 5 min after each injection and final injection of 300 µL of regeneration buffer (PBS, pH 7.4, 1 M NaCl, 0.03% Tween and 0.5% glycerol) and 300 µL of PBST. The chips were used to up to 24 times without significant loss of capturing capacity.

### 2.5. Interaction of GPLD1 and Serum Proteins with Micelle-Like bAChE Complexes

Principle: Three different modes for capture by the chip surface of bAChE complexes, TX-100 bAChE micelles, or bAChE liposomes (injection of 75 µL of suspension containing identical AChE volume activity at a flow rate of 15 µL/min), which had been prepared as described previously [29], as putative interaction partners for GPLD1 as well as other serum proteins were used: For covalent capture via the protein moiety of bAChE, the microfluidic channels of uncoated chips were primed by three injections of 150 µL each of immobilization buffer at a flow rate of 45 µL/min. Then the chip surface was activated by a 150 µL injection of 0.2 M EDC and 0.05 M Sulfo-NHS (mixed from 2x-stock solutions right before injection) at a flow rate of 45 µL/min. After a waiting period of 3 min, subsequent injection of the suspension, an additional waiting period (flow rate 0) for 2 min and final washing of the channels with 300 µL of PBST at a flow rate of 180 µL/min, the residual activated groups on the chip surface were capped by injecting 200 µL of 1 M ethanolamine (pH 8.5) at a flow rate of 60 µL/min. For capture by α-toxin via the GPI glycan core of bAChE, α-toxin-coated plain AU chips were used as described previously [29]. For ionic capture, uncoated negatively charged and highly hydrophilic TiO_2_ chips were used. TiO_2_ repels negatively charged phospholipids. Hence, the immobilization of micelle-like bAChE complexes and bAChE liposomes containing positively charged, negatively charged, or zwitterionic phospholipids or combinations thereof at the surface of TiO_2_ chips with high efficacy was performed in the presence of 2 mM Ca^2+^, 10 mM Hepes/NaOH (pH 7.5), and 100 mM NaCl to enable salt bridges between the chip surface and the phospholipids [42]. The measured phase shift for binding of bAChE liposomes was found to be considerably higher for TiO_2_-Ca^2+^ compared to Au and SiO_2_ surfaces (data not shown). Ca^2+^ easily covers the TiO_2_ surface, forming a complete interactive layer. Thus, the phospholipids of the liposomal membranes and micelle-like complexes can bind to many sites on the surface at high density. High amounts were found to be bound to the TiO_2_ surface, indicating that close to complete coverage had been achieved. In contrast, Au and SiO_2_ surfaces were only partially covered, presumably due to repulsive forces between the bound liposomes or complexes, while other parts of the chip surface remained free of phospholipids (“mosaic”; data not shown). In addition, the presence of Ca^2+^ during the injection may prevent the repulsion between individual bAChE liposomes or micelle-like bAChE complexes and trigger the fusion of liposomes and micelles, respectively. Thus, capture of bAChE liposomes and micelle-like bAChE complexes by the TiO_2_ chip surface presumably led to their transformation into flat supported membrane bilayers. With time, the membrane and micelle layers, formed via the unstably bound Ca^2+^, tended to detach from the surface (“bleeding”; data not shown).

General procedure: For each mode of capture, the chips were washed with 2 × 200 µL of PBST at a flow rate of 120 µL/min prior to injection of 150 µL of serum, which had been diluted 20-fold with PBS, at a flow rate of 14 µL/min (for routine assay with 1× serum, for other experiments with 2× and 5× serum and flow rates adjusted to 28 and 70 µL/min, respectively). After washing with 2× 180 µL at a flow rate of 120 µL/min, 150 µL of anti-GPLD1 antibody, diluted 1000-fold with PBS, were injected at a flow rate of 15 µL/min. Following an additional washing step, 125 µL of 30 µM PIG41 were injected at a flow rate of 15 µL/min. Alternatively, 75 µL of anti-AChE antibody, diluted 500-fold with PBS, were injected at a flow rate of 15 µL/min. For regeneration of the chips, 250 µL of 0.3% Triton X-100, 10 mM glycine (pH 12) were injected.

### 2.6. Isolation of HDLs (High-Density Lipoproteins) from Human Plasma

Venous blood from adult volunteers (after 12–14 h overnight fast) was collected into tubes (Vacutainer, Becton-Dickinson, Franklin Lakes, NJ, USA) containing disodium EDTA (1.5 mg/mL). Plasma was promptly separated by low-speed centrifugation (1000× *g*, 10 min, 4 °C) and then passed through dextran sulfate cellulose. HDLs (1.063 < d < 1.210 g/mL) were isolated from frozen plasma samples of normolipidemic humans (for the preparation of HDL reconstituted with hCD73 (hCD73-recHDL)) or of type 1/type 2 patients and control probands (for the measurement of HDL concentration) by serial ultracentrifugation as published previously [43,44,45]. The density of the plasma was raised by addition of NaCl and KBr and checked by pyknometry at 20 °C [46] with the following modifications: 2 mL of 0.196 M NaCl were layered on top of 4 mL of human plasma and centrifuged (300,000× *g*, 24 h, 4 °C), and 0.2 mL of fractions were collected from the top. Under these conditions, very low density lipoproteins (VLDL) and chylomicrons floated to the top and were removed. The remaining fractions (lacking VLDL and chylomicrons) were combined and then diluted with 0.196 M NaCl to a final volume of 3.3 mL, and 660 µL of 4.778 M NaBr were added. Following intense mixing, 2 mL of 0.844 M NaBr were layered on top and then centrifuged (300,000× *g*, 24 h, 4 °C). Under these conditions, low density lipoproteins (LDL) floated to the top and were removed. The remaining fractions (lacking VLDL, chylomicrons, and LDL) were diluted with 2.65 mL of 0.844 M NaBr and then supplemented with 1.34 mL of 7.593 M NaBr. After mixing, and subsequent overlaying with 2 mL of 2.973 M NaBr, subsequent centrifugation (300,000× *g*, 48 h, 4 °C) led to flotation of the HDLs onto top. After dialysis against PBS (pH 7.4), 1 mM EDTA, and concentration (if necessary), HDLs were assayed for cholesterol, apo A-I, AChE, CD73, and CD55.

### 2.7. Reconstitution of HDL Harboring Human CD73 (hCD73-recHDL)

The hCD73-recHDL was reconstituted by the cholate dialysis method. In brief, 0.5 g of dioleoylphosphatidylcholine and 0.5 g of dipalmitoylphosphatidylcholine were solubilized in chloroform in glass tubes, dried in a stream of nitrogen, and then resuspended in 300 μL of 150 mM NaCl, 0.1 mM EDTA (pH 7.5). The lipid mixture was vortexed thoroughly and stored on ice (60 min). After addition of 350 µL of sodium cholate solution and 1.2 mg HDLs, the mixture was intensely vortexed every 10 min for 75 min until complete clearance of the solution had been achieved. Then, 1.5 mg of lyophilized apo A-I (prepared and purified by fast performance liquid chromatography from delipidated HDL as described previously [47]) and 300 µg of hCD73 in 0.1% ß-amidotaurocholate (BATC) were added to the lipid mixture in Eppendorf tubes and then incubated (120 min, on ice). Thereafter, the mixture was dialyzed with PBS (pH 7.5), 0.1 mM EDTA for 16 h at 4 °C using dialysis tubings with 50 kDa cut-off. The hCD73-recHDL was washed by flotation (300,000× *g*, 48 h, 4 °C) at d = 1.210 g/mL, gel-filtered on high resolution Superose 6B column (1 × 30 cm, LKB/Pharmacia, Uppsla, Sweden), and then eluted with TBS at a flow rate of 0.5 mL/min. Protein concentration was followed by absorbance at 280 nm. Then, 0.5 mL fractions were collected and quantitatively assayed for the presence of cholesterol and hCD73 by dot blotting. Fractions exhibiting both cholesterol and hCD73 were pooled and subjected to immune adsorbent purification (batch procedure). Material bound to the anti-apo A-I immune adsorbent was eluted with two to three (immune adsorbent) volumes of 0.1 M glycine/HCl (pH 2.8), immediately buffered to pH 7.4 with solid Tris, then dialyzed against PBS (pH 7.4), 1 mM EDTA, and after concentration under vacuum (Micro-Confilt concentrator) to 1 mL, stored at 4 °C.

### 2.8. Translocation of bAChE from Micelle-Like Complexes, Liposomes, and HDLs to Rat Adipocytes

The protocol encompasses four steps: (i) the translocation per se in the course of an initial incubation of primary rat adipocytes with micelle-like complexes, liposomes, or HDLs reconstituted with bAChE or hCD73 in the absence or presence of serum proteins, (ii) the recovery of the adipocytes harboring the translocated bAChE or hCD73 by centrifugation of the incubation medium through dinonylphtalate, (iii) the spontaneous release of the translocated bAChE or hCD73 from the washed adipocytes in the course of a subsequent incubation as a measure for bAChE or hCD73 translocated during the initial incubation, and (iv) the measurement of the amount of released (and initially translocated) bAChE or hCD73 by chip-based sensing. (ad i) Primary rat adipocytes were prepared from epididymal fat pads of male Wistar rats (140–160 g, fed *ad libitum*) as described previously [28], thereafter suspended in 2.5 mL of adipocyte buffer (20 mM Hepes/KOH, pH 7.4, 140 mM NaCl, 4.7 mM KCl, 2.5 mM CaCl_2_, 1.2 mM MgSO_4_, 1.2 mM KH_2_PO_4_, 2% [*w*/*v*] BSA, 100 μg/mL gentamycin, 1 mM sodium pyruvate, 5.5 mM glucose) at a lipocrit of 0.1% and then incubated (under conditions as indicated in the figures) with various amounts of micelle-like complexes, liposomes or recHDL containing (or lacking) bAChE or hCD73 (prepared as described in [29] and the Supplementary Materials) in the presence or absence of serum samples (diluted 10-fold with PBS) in 20 mL plastic vessels in a shaking water bath (100 cycles/min) under constant bubbling with 5% CO_2_/95% O_2_. (ad ii) Thereafter, three 350-µL portions of the total mixtures were transferred to microfuge tubes (Beckman Inc., Krefeld, Germany) prefilled with 100 µL of dinonylphtalate and then centrifuged (1000× *g*, 1 min, 20 °C). The tubes were cut through the dinonylphtalate layer separating the incubation medium in the bottom part of the tubes from the adipocytes at the top part. Both parts were rescued, taking care to minimize the volume of dinonylphtalate taken along with the adipocytes and incubation medium, respectively. The bottom parts were assayed for lactate dehydrogenase (LDH) activity, and the top ones for the amount of translocated bAChE or hCD73. For this, 50 µL portions of the adipocytes were transferred to 0.5 mL Eppendorf cups. After addition of 450 µL of adipocyte buffer, gentle mixing, centrifugation (1000× *g*, 1 min, 20 °C), and subsequent suction of the infranatant, 450 μL of adipocyte buffer was added to the cells. (ad iii) Then, 30 µL portions of the washed adipocytes were supplemented with 470 µL of adipocyte buffer and then incubated (4 h, 30 °C) in 10 mL plastic vessels in a shaking water bath (100 cycles/min) under constant bubbling with 5% CO_2_/95% O_2_. Thereafter, 350 µL portions of the total mixtures were transferred into microfuge tubes (Beckman), prefilled with 100 µL of dinonylphtalate, and then centrifuged (1000× *g*, 1 min, 20 °C). The tubes were cut through the dinonylphtalate layer, separating the adipocytes at the top from the incubation medium (adipocyte-free infranatant) in the bottom part of the tubes, which was rescued. Care was taken to minimize the volume of dinonylphtalate taken along with the infranatant in the bottom part. (ad iv) After transfer of the adipocyte-free infranatant (300 µL) to new cups, addition of 200 µL of adipocyte medium containing 1 mM DTT, 2-fold protease inhibitor mix, and subsequent centrifugation (10,000× *g*, 10 min, 4 °C) for removal of any particulate materials (cell debris), 450 µL of the supernatant (cleared adipocyte-free infranatant) was injected into α-toxin coated chips at a flow rate of 60 µL/min. After washing of the channels with 500 µL of running buffer at a flow rate of 200 µL/min, 100 µL of annexin-V in the presence of 2 mM Ca^2+^, 100 µL of anti-AChE (1:500), 90 µL of anti-rCD73 (1:1000), 85 µL of anti-hCD73 (1:500), 70 µL of anti-CD55 (1:2000), and 60 µL of anti-aP (1:200) antibodies were injected in sequential fashion at a flow rate of 15 µL/min with washing cycles with 450 µL of running buffer at a flow rate of 200 µL/min between two injections each. Finally, 50 µL of 30 µM PIG41 were injected at a flow rate of 15 µL/min.

### 2.9. Effect of Micelle-Like bAChE Complexes on LDH Release of Rat Adipocytes

Primary rat adipocytes were incubated with micelle-like bAChE complexes as described above for the determination of bAChE translocation (see i) and then separated from the incubation medium by centrifugation through dinonylphtalate (see ii). The incubation medium was then assayed for LDH activity according to Zou and coworkers [48] with the following modifications: 300 µL of the incubation medium was transferred to 1.5 mL microcentrifuge tubes and centrifuged (12,000× *g*, 15 min, 4 °C) for removal of any cell debris. LDH activity was measured by a two-step reaction encompassing oxidation of lactate to pyruvate under concomitant reduction of NAD^+^ to NADH_2_ and subsequent reduction of nitroblue tetrazolium to methyl hydrazine by diaphorase and NADH_2_. The amount of methyl hydrazine generated, which was detected by absorbance measurement at 560 nm, was proportional to the amount of LDH. For this, 50 µL portions were transferred to the wells of flat-bottom 96-well plates (Becton-Dickinson, Franklin Lakes, NJ, USA), and then mixed with 50 µL of 10 mM Tris/HCl (pH 8.5), 0.444 mg/mL nitro blue tetrazolium, 0.54 mg/mL NAD^+^, 20 U/mL diaphorase, and 50 mM lactic acid (lithium salt). After incubation (30 min, 20 °C), 10 µL of 1 M HCl were added to each well to terminate the reaction. The absorbance of the color that developed and reflected LDH activity was determined at 560 nm using a microplate reader (MPR-A4i, TOSOH, Tokyo, Japan). As control for nonspecific release, the spontaneous release of LDH from adipocytes, which had not been incubated in primary culture but subjected to the same procedure for separation from the incubation medium, was determined and subtracted as “mechanically” induced nonspecific release from each value.

### 2.10. Reconstitution of Erythrocyte Band-3 Protein into Liposomes

Stripped erythrocyte ghosts from rabbit blood were prepared as described previously [49]. The final pellet was suspended in 5 mM sodium phosphate buffer (pH 7.4) and stored at −80 °C. For reconstitution of band-3 into liposomes, a mixture of lipids at 25-fold excess (*w*/*w*) to stripped erythrocyte ghosts was subjected to cycles of freezing/thawing and sonication [49]. For this, the lipids (dissolved in chloroform) were first dried under argon and resuspended in 10 mM Mes/KOH (pH 5.5), 2 mM MgSO_4_, and 20 mM Na_2_SO_4_ in a final volume of 125 mL. After 15 successive 1 s sonication cycles using a bath sonicator (model 2200; Branson Ultrasonic Corp., Danbury, CT, USA), the lipids were mixed with stripped erythrocyte ghosts (50 mg protein) in a final volume of 150 mL. This reconstitution mixture was subjected to one cycle of rapid freezing in liquid nitrogen and thawing at room temperature, followed by 30 s of sonication in the bath sonicator.

### 2.11. Pretreatment of Serum

Heat: Serum samples were incubated in 1 mL Eppendorf cups (15 min, 65 °C), cooled on ice, and then centrifuged (10,000× *g*, 2 min, 4 °C) for removal of any precipitated materials. The supernatant was stored at −80 °C. Protease: 45 µL of serum (diluted 10-fold with PBS) were incubated (90 min, 4 °C) in the presence of 5 µL of proteinase K (1 mg/mL in H_2_O). For termination, 2.5 µL of PMSF (100 mM in isoproterenol) were added and the incubation continued (5 min, 4 °C). PEG precipitation: 50 µL of serum were supplemented with 50 µL of 12% (*w*/*v*) PEG6000 (in H_2_O) and then incubated (4 h, 4 °C). After centrifugation (10,000× *g*, 20 min, 4 °C), the supernatant was transferred to new tubes and stored at −80 °C. Immune depletion of GPLD1: 20 µL of serum were supplemented with 180 µL PBS in 0.5 mL Eppendorf cups. After addition of 2 µL (2 µg) anti-GPLD1 antibody and incubation (2 h, 4 °C), 20 µL of protein A/G-Sepharose (10% (*w*/*v*), preincubated in PBS, 0.1 mM EDTA for 60 min) were added and the incubation continued (20 h, 4 °C, head-over-tail rotation). After centrifugation (10,000× *g*, 5 min, 4 °C), the supernatant was taken off and then spun again under identical conditions. The supernatant was transferred to new vials and stored at −80 °C.

### 2.12. GPLD1 Treatment of Micelle-Like bAChE Complexes

A total of 20 µL of micelle-like bAChE complexes were supplemented with 20 µL of 20 mM Hepes/KOH (pH 7.0), 300 mM NaCl, 2 mM Ca^2+^ and then incubated (1 h, 37 °C) with 15 ng GPLD1, which had been purified from bovine serum by sequential chromatography on anti-GPLD1 antibody, wheat germ lectin, and monoQ columns according to [36].

### 2.13. Statistical Analysis

All numerical data were presented as means ± standard deviations (SDs). Statistical significance was calculated using GraphPad Prism6 software (v6.0.2, GraphPad Software, San Diego, CA, USA) on the basis of either the two-tailed unpaired Student’s *t*-test between two experimental groups or the one-way ANOVA performed with Tukey’s multiple comparison post-test. *p* ≤ 0.05 was considered to be significant. Linear regression analysis of primary data was performed with FitMaster Origin-based software (OriginLab Inc., Northampton, MA, USA).

### 2.14. Miscellaneous

Chemical synthesis of PIG41, protein determination, preparation of primary rat adipocytes, assay for AChE enzymic activity, preparation of α-toxin from the culture supernatant of *Clostridium septicum* and bAChE from bovine erythrocytes, coupling of α-toxin to the gold chip surface of long-chain 3D CM-dextran sam^®^ 5 chips using conventional EDC/NHS-based protocol in a SamX instrument (SAW/Nanotemper, Bonn/Munich, Germany), and SAW measurement, instrumentation and evaluation were performed as has been described in detail previously [28,29,30,50]. Before injection of serum samples into CM-dextran chips, 0.1 vol. of 10 mg/mL carboxymethyl dextran (sodium salt, 0.15 M NaCl, 0.02% NaN_3_ (NSB reducer) was injected in order to reduce nonspecific binding of sample components to the chip surface through competition without affecting the measurement of specific interactions.

## 3. Results

### 3.1. Strong and Weak Dependence on Metabolic Derangement of Serum GPI-PLD Activity and GPLD1 Amount, Respectively, in Rats

Full-length GPI-APs, such as bAChE from bovine erythrocytes, reconstituted together with (lyso)phospholipids and cholesterol into micelle-like complexes at an “optimized” constituent ratio, were demonstrated to represent preferred substrates for degradation by serum GPI-PLD activity [30]. So far, in mammalian serum this activity has been attributed to GPLD1 exclusively [51]. Using that mode of substrate presentation, the upregulation of GPLD1 activity in response to metabolic derangement was measured for six cohorts (eight animals each) of normal and metabolically deranged rats (Table 1).

GPLD1 activity was measured as the phase shift upon capture of the cleaved hydrophilic bAChE protein moiety, having lost the phosphatidate but retained the inositolglycan portions of the GPI anchor, by α-toxin-coated chips. The specificity of the capture was assayed with control injections of excess of PIG41, which acts as competitor for the interaction between the GPI anchor and α-toxin [39,52]. GPLD1 activity was found to vary within the groups and to significantly differ between the groups (Figure S1). Obese hyperinsulinemic hyperglycemic ZDF rats exhibited the highest activity, followed by obese hyperinsulinemic normoglycemic ZF and obese mildly hyperinsulinemic normoglycemic Wistar rats, and lean normoinsulinemic normoglycemic Wistar rats expressed the lowest activity (Figure S1a–c). Calculation of the means (eight rats *per* group) of the specific phase shifts as the difference between the maximal phase shift and the unspecific one (left in the presence of excess of PIG41) revealed significant differences between the groups with the exception of obese Wistar and ZF rats (Figure S1d). Strikingly, GPLD1 activity measurement by chip-based sensing enabled the differentiation of lean Wistar, lean normoinsulinemic normoglycemic ZF, and lean mildly hyperinsulinemic normoglycemic ZDF rats, which are characterized by similar blood glucose in conjunction with no (Wistar, ZF) or only mild elevation of plasma insulin level (ZDF) (Table 1). These data confirmed the results of a previous study that had been obtained with different animals belonging to the same six categories of genotype and nutritional state [30]. The presence of the Ca^2+^-chelating agent Pha reduced the serum-induced specific phase shift by more than 90% (Figure S1d), which is compatible with the known Ca^2+^-dependence of GPLD1 [37,38] and the specificity of the assay.

**Figure 1:**
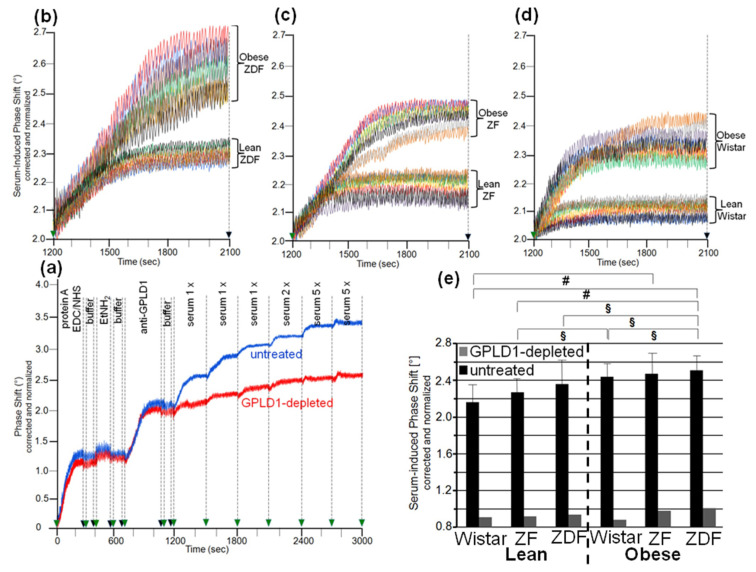
Measurement of the amount of GPLD1 in rat serum using chip-based assay. (**a**) For setup of the assay, first protein A was covalently immobilized onto EDC/NHS-activated chips. Following washing with buffer and blockade of unreacted carboxyl groups by injection of ethanolamine (EtNH_2_), anti-GPLD1 antibody was injected into the channels. After washing of the chips, single or multiple (as indicated) 100 µL portions of serum (diluted 10-fold) from obese ZDF rats, which had been depleted of GPLD1 by immune adsorption (red curve) or remained untreated (blue curve), were injected successively. The experiment was repeated once (distinct chip) with similar results (representative shown in **a**). The measured phase shift is given upon correction for unspecific interaction of serum components (protein A-coated control channel lacking anti-GPLD1 antibody) and altered viscosity (vs. buffer) of the sample fluid and normalization for the varying responsiveness of distinct chips toward GPI-AP capture. (**b**–**d**) Identical volumes (single injection of three 100 µL portions) of sera were injected into chips with bound anti-GPLD1 antibody at time point 1200 s (repeated three times with different chips) with similar results (representatives shown). The phase shift measured as described for (**a**) is shown for the period of serum injection only (serum-induced phase shift: 1200–2100 s). (**e**) The serum-induced phase shift determined in the absence of anti-GPLD1 antibody (*n* = 8; means ± SD, quadruplicate measurements, black bars) and its presence (*n* = 1; duplicate measurements, grey bars) is given. The comparison between the six rat groups is shown for the sera not subjected to immune adsorption (black bars) only (^#^ *p* ≤ 0.02, ^§^ *p* ≤ 0.05).

The differences in GPLD1 activity between the rat groups may rely on its differential expression. For assessment of the amount of GPLD1 protein by chip-based sensing, the serum samples were injected into channels that had been coated with anti-GPLD1 antibody via covalently coupled protein A, as indicated by the stepwise increases in the phase shift upon the corresponding injections (Figure 1a). The successive injection of 10 μL portions of diluted serum from obese ZDF rats at an increasing number (blue curve) caused further elevations of the phase shift, demonstrating the (volume-dependent) detection of GPLD1 in (1–20 μL of undiluted) rat serum. The specificity for GPLD1 was confirmed by the decline in phase-shift increases in the course of injection of GPLD1-depleted serum (red curve).

Analysis of serum samples from eight individual rats *per* group for GPLD1 protein revealed variance in the serum-induced phase shift within the groups and significant differences between the groups. Obese ZDF rats expressed the highest amount (Figure 1b), followed by obese ZF (Figure 1c) and obese Wistar rats (Figure 1d), and finally lean Wistar rats (Figure 1d). Calculation of the means of serum-induced phase shift resulted in significant differences between certain groups, and was most prominent for obese ZDF and lean Wistar rats (Figure 1e, black bars). However, the potency of differentiation was lower with GPLD1 amount compared to activity, as the former did not differ between lean Wistar, ZF, and ZDF, or between obese Wistar and ZF, or between obese ZF and ZDF rats. The drastic reduction of the serum-induced phase shift by use of GPLD1-depleted serum demonstrated the specificity of chip-based sensing of GPLD1 protein (Figure 1e, grey bars).

### 3.2. Dependence of the Interaction of Serum GPLD1 with Micelle-Like bAChE Complexes on Metabolic Derangement in Rats and Humans

The loss of differentiation power for the rat groups when measuring serum GPLD1 protein (Figure 1) compared to activity (Figure S1) raised the possibility that the portion of GPLD1 interacting with and degrading the anchor is not reflected by the total amount of GPLD1 protein in serum. Therefore, the amount of serum GPLD1 interacting with micelle-like bAChE complexes was determined. For stabilization of the interaction in vitro, the assay was performed in presence of the Ca^2+^-chelator Pha for inhibition of the Ca^2+^-dependent enzyme [36]. For determination of GPLD1 associated with micelle-like bAChE complexes by chip-based sensing, the complexes were captured by the chip channels using three distinct modes, (i) capture by α-toxin via the bAChE core glycan (Figure S2a,b), (ii) ionic capture through Ca^2+^ bridges between the negatively charged TiO_2_ chip surface and the negatively charged (lyso)phospholipids of the complexes (Figure S2c,d), and (iii) covalent capture via the bAChE protein moiety (Figure S2e,f).

**Figure 2:**
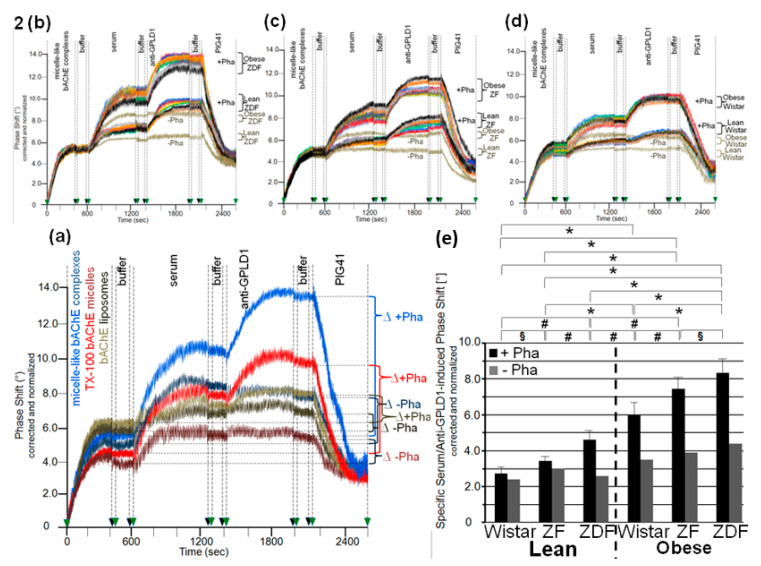
Measurement of the interaction of GPLD1 and other proteins from rat serum with micelle-like bAChE complexes. (**a**–**d**) bAChE reconstituted into micelle-like complexes at the “optimized” constituent ratio, liposomes (**a** only), and TX-100 micelles (**a** only) were injected into α-toxin-coated chips. Following washing of the chips with buffer, identical volumes of serum from obese ZDF rats (**a**) or from the six rat groups as indicated (**b**–**d**) were injected in the presence or absence of Pha (0.5 mM) as indicated. After washing with buffer, anti-GPLD1 antibody, buffer, and PIG41 (30 µM) in the presence or absence of Pha (corresponding to the serum injection) were injected in sequential fashion. Correction and normalization of the phase shift were performed as described for Figure 1. (**a**) The total combined serum- and anti-GPLD1 antibody-induced phase shift is indicated as the difference (∆) between termination of antibody injection with subsequent washing (2100 s) and termination of the coating of the chips with subsequent washing prior to injection of serum (600 s). (**b**–**d**) Comparisons between the six rat groups were performed with the same chip each and repeated three times (different preparations of micelle-like bAChE complexes) with similar results (16 regeneration cycles *per* chip; representatives shown). (**e**) The specific combined serum- and anti-GPLD1 antibody-induced phase shift was calculated as the difference between the total phase shift (see **a**) and the unspecific phase shift remaining left at the end of PIG41 injection (2600 s). The phase shifts determined in the presence of Pha (*n* = 8; means ± SD, triplicate measurements, black bars) or absence of Pha (*n* = 1; duplicate measurements, grey bars) are given. The comparison between the groups is shown for the samples coinjected with Pha (black bars) only (* *p* ≤ 0.01, ^#^ *p* ≤ 0.02, ^§^ *p* ≤ 0.05).

Identical amounts of bAChE reconstituted into micelle-like complexes, liposomes, or TX-100 micelles were captured by α-toxin-coated chips, which led to similar increases in phase shift (Figure 2a). Injection of serum from obese ZDF rats together with Pha (light blue curve) led to further elevation in the phase shift, which was considerably higher for micelle-like complexes compared to TX-100 micelles (light red curve) and liposomes (light brown curve). For both complexes and micelles, but not liposomes, the phase-shift increases were considerably diminished but not completey abrogated in the absence of Pha (Figure 2a, dark-colored curves). This argued (i) that serum GPLD1 interacts with bAChE when presented in micelle-like complexes or TX-100 micelles, that (ii) this interaction is prone to detection upon blockade of GPLD1 activity, and that (iii) serum components other than GPLD1 interact with bAChE as well. Subsequent injection of anti-GPLD1 antibody led to high, medium and low phase-shift increases for micelle-like bAChE complexes, TX-100 bAChE complexes, and bAChE liposomes, respectively, but only in the presence of Pha, confirming the above conclusions (i) and (ii) (Figure 2a). Reduction of the phase-shift increase upon injection of PIG41 demonstrated the specificity of the capture, as well as interaction of GPLD1 and other serum components, with bAChE. Consequently, in the following the interaction of full-length GPI-APs with serum components was assayed with micelle-like bAChE complexes captured by α-toxin-coated chips and coinjection of Pha, which yielded the highest serum- and anti-GPLD1-induced phase-shift increases.

The serum- and anti-GPLD1-induced phase shifts (eight individual rats *per* group) revealed variance within the groups and significant differences between the groups. Obese ZDF and lean Wistar rats exhibited the most pronounced and lowest shifts, respectively (Figure 2b–d). Strikingly, omission of Pha caused lowering and complete abrogation of the serum- and anti-GPLD1 antibody-induced phase shifts, respectively, for each group. Loss of phase shift by final injection of PIG41 demonstrated specificity of the capture of micelle-like bAChE complexes, as well as of the interaction of serum components and GPLD1 with the complexes (Figure 2b–d).

The means of the serum- and anti-GPLD1 antibody-induced specific phase shifts revealed significant differences between all groups, and were most prominent between lean and obese rats of the same genotype (Figure 2e, black bars). The reduction of the serum- and anti-GPLD1 antibody-induced phase shifts by the absence of Pha for each group (Figure 2e, grey bars) is explained best by cleavage of bAChE by GPLD1 resulting in their dissociation in the presence of Ca^2+^. However, the maintenance of the residual phase shift and its dependence on the metabolic derangement of the rats in the absence of Pha reemphasized that in addition to GPLD1, other bAChE-interacting components are expressed in serum from rats. Taken together, serum components, among them GPLD1, interact with micelle-like GPI-AP complexes depending on the genotype (Wistar, ZF, ZDF) and nutritional state (lean, obese).

**Figure 3:**
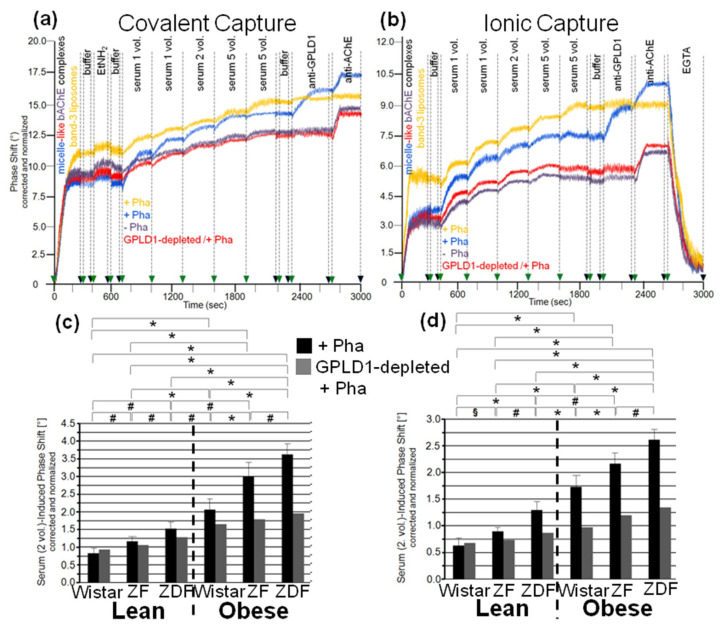
Measurement of the interaction of GPLD1 and other rat serum proteins with micelle-like bAChE complexes using covalent or ionic capture by the chips. Micelle-like bAChE complexes (blue, red, purple curves) or band-3 liposomes (orange curves), respectively, were covalently captured by chips upon injection into EDC/NHS-activated chips, washing with buffer, blockade of unreacted carboxyl groups by injection of EtNH_2_, and additional washing with buffer (**a**,**c**) or non-covalently captured by injection of Ca^2+^ and subsequent washing with buffer (**b**,**d**). (**a**,**b**) Single or multiple 100 µL portions (as indicated) of serum from obese ZDF rats, which had been depleted of GPLD1 by immune adsorption (red curves) or remained untreated (blue, purple, orange curves), were injected successively in the absence (purple curves) or presence (blue, red, orange curves) of Pha (0.5 mM). Following washing with buffer, anti-GPLD1 and anti-AChE antibodies were injected in sequential fashion. Finally, as a test for capture of the micelle-like bAChE complexes via Ca^2+^ bridges, EGTA (5 mM) was injected (shown only for **b**). The experiment was repeated once (distinct chip) with similar results (representatives shown). The measured phase shift is given upon correction for unspecific interaction with phospholipids (use of micelle-like complexes lacking protein) and altered viscosity (vs. buffer) of the sample fluids and normalization for the varying responsiveness of distinct chips toward GPI-AP capture. (**c**,**d**) Two 100 µL portions each of untreated and GPLD1-depleted serum were injected. The serum-induced phase shift was calculated as the difference between time points 700/400 s (end of capture and washing) and 1300/1000 s (end of serum injection) for both covalent (**c**) and ionic (**d**) capture, and is given for the Pha-treated (*n* = 8; means ± SD, quadruplicate measurements, black bars) and GPLD1-depleted as well as Pha-treated (*n* = 1; duplicate measurements, grey bars) sera. The comparison of the serum-induced phase shift between the six rat groups is shown for the Pha-treated samples (black bars) only (* *p* ≤ 0.01, ^#^ *p* ≤ 0.02, ^§^ *p* ≤ 0.05).

To exclude an impact of capture of the micelle-like bAChE complexes by α-toxin on their interaction with serum components, covalent capture of the bAChE protein moiety (Figure 3a) and ionic capture of the complex phospholipids (Figure 3b) were performed. The efficacy of these alternative capture modes was demonstrated by the considerable phase-shift increases following injection of anti-AChE antibody (Figure 3a,b) and their abrogation by final injection of EGTA in case of ionic (Figure 3b), but not covalent capture (data not shown). On the basis of identical amounts of micelle-like bAChE complexes injected (Figure 3a,b, blue curves), covalent capture was more efficient than the ionic one. This held also true for liposomes reconstituted with the erythrocyte transmembrane protein band-3 (orange curves). Those were captured with higher efficacy compared to the complexes. Injection of 10-μL portions of diluted serum from obese ZDF rats at an increasing number (corresponding to 2–28 μL of undiluted serum) together with Pha into the chips with captured complexes caused further elevations of the phase shift. This reflected the interaction of serum components with micelle-like bAChE complexes. With both capture methods, the incremental increases in the phase shift declined with the number of injection cycles, i.e., amount of serum components, demonstrating saturation of the interaction (Figure 3a,b). In contrast, the phase shifts induced by band-3 liposomes, containing the same amount of bAChE as the complexes, were considerably lower compared to the complexes at the lowest amount, but did not decline with subsequent injection cycles (orange curves). The observed and missing volume-dependence and saturation of the phase-shift increments, respectively, argued for the specific and nonspecific nature of the interaction of serum components with micelle-like bAChE complexes and band-3 liposomes, respectively.

**Figure 4:**
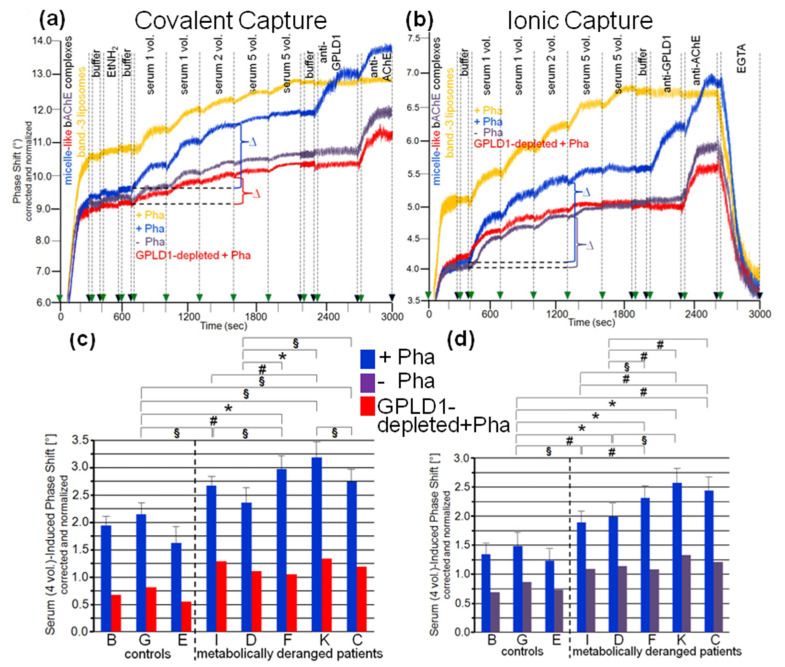
Measurement of the interaction of GPLD1 and other human serum proteins with micelle-like bAChE complexes using covalent (**a**,**c**) or ionic (**b**,**d**) capture by the chips. The experiment was performed as described for Figure 3 using pooled serum from the controls (**a**,**b**) and individual sera from the controls and metabolically deranged patients (**c**,**d**). (**a**,**b**) The serum-induced phase shift is calculated as the differences (∆) measured before (at 700/400 s) and after sequential injection (at 1600/1300 s) of four 100-µL portions of serum (which guaranteed nonsaturation of the chips for GPI-APs). (**c**,**d**) The serum-induced phase shift of the individual samples determined in the presence (quadruplicate measurements, blue bars, means ± SD) (**c**,**d**) and absence (single measurement, purple bars) of Pha (**d**) or upon depletion of GPLD1 by immune adsorption in the presence of Pha (single measurement, red bars) (**c**) is given. The comparison between the individual probands is shown for the samples treated with Pha (blue bars) only (* *p* ≤ 0.01, ^#^ *p* ≤ 0.02, ^§^ *p* ≤ 0.05).

With both capture modes, the omission of Pha during the injections (purple curves) or the use of GPLD1-depleted serum (red curves) caused reductions in the serum-induced phase-shift increments by 30 to 40% (Figure 3a,b). Successful removal of GPLD1 from serum was confirmed by failure of anti-GPLD1 antibody to elicit further phase-shift increase (red curves), at variance with native serum (blue curves). Moreover, the anti-GPLD1 antibody failed to induce phase-shift increase with band-3 liposomes, compatible with failure of GPLD1 to interact with liposomal phospholipids (orange curves). Importantly, very similar results were obtained with serum from control probands (Table 2) and the use of both covalent (Figure 4a) and ionic (Figure 4b) capture of the micelle-like bAChE complexes. Together, these findings suggested that GPLD1 and other components from rat and human serum interact with micelle-like bAChE complexes in a specific fashion.

Analysis of the specific interaction between micelle-like bAChE complexes and rat serum components in the presence of Pha (Figure 3c,d, black bars) revealed significant differences in the serum-induced phase-shift increases (with four volume equivalents) between the groups, most prominent between lean and obese rats of the same genotype. The absence of GPLD1 (in the presence of Pha) (Figure 3c,d, grey bars) diminished the serum-induced phase-shift increase by up to 70% with obese rats. This indicated that in serum of obese rats, the major portion of the components interacting with micelle-like bAChE complexes is constituted by GPLD1. The maintenance of the residual phase shift in the absence of GPLD1 protein or activity and its dependence on the metabolic derangement of the rats reinforced the presence of serum components interacting with GPI-APs and being different from GPLD1.

Analysis of the specific interaction between micelle-like bAChE complexes and human serum components in the presence of Pha (Figure 4c,d, blue bars) led to differentiation between the three healthy controls and the five metabolically deranged patients (Table 2). Each of the latter exhibited a significantly higher serum-induced phase-shift increase (with four volume equivalents) compared to the control “G”, displaying the highest increase among the controls. Moreover, significant differences in serum-induced phase-shift increase were apparent within the metabolically deranged patients, but not within the controls (Figure 4c,d, blue bars). Thus, measurement of the specific interaction of serum components with full-length bAChE by chip-based sensing using both capture modes enabled differentiation of healthy and metabolically deranged humans. The absence of Pha (Figure 4d, purple bars) or GPLD1 (in the presence of Pha) (Figure 4c, red bars) led to reduction of the serum-induced phase-shift increase by 45 to 85%. This indicated that more than half of the human serum components interacting with micelle-like bAChE complexes is constituted by GPLD1. The maintenance of the residual phase shift and its dependence on metabolic derangement vs. control in the absence of GPLD1 activity or protein confirmed the presence of serum components in humans interacting with GPI-APs and being different from GPLD1.

It has been amply documented that in serum, GPLD1 binds to HDLs, which triggers dissociation of the high-molecular complexes formed by this amphiphilic protein in the absence of detergent [53]. The interaction between GPLD1 and HDLs is mediated by its major component, apolipoprotein A-I [54] and leads to robust stimulation of cleavage of detergent-solubilized GPI-APs by the GPLD1-HDL-apo A-I complex in vitro [55]. Therefore, the possibility was evaluated that part of the serum GPLD1 activity measured with micelle-like bAChE complexes is bound to HDLs, which thereby supports cleavage of substrate GPI-APs upon their fusion with the GPLD1-HDL-apo A-I complexes. In fact, residence of the GPI-AP CD59 at HDL particles in human plasma was reported previously [27,56], thus demonstrating the possibility of anchorage of GPI-APs at (the phospholipid monolayer) of lipoproteins. This finding was corroborated in the present study by positive dot blotting of HDLs isolated from the eight human plasma samples for CD73, CD55, and AChE, albeit the amounts determined were rather low and considerably differed between the three GPI-APs (Table 3a). Importantly, only trends for higher and lower HDL-cholesterol in plasma of type 1 and type 2 diabetic patients, respectively, compared to control probands were measured (Table 3a). These findings were in agreement with clinical data available for (i) type 2 diabetic patients, that the higher the plasma insulin level, the lower the plasma HDL-cholesterol and apo A-I concentrations, and that consequently both of them are typically decreased compared to healthy probands, as well as for (ii) type 1 diabetic patients treated with insulin, that their plasma HDL-cholesterol and apo A-I concentrations are not lower than normal, and even tend to be higher than those in healthy probands [57,58,59,60,61,62,63]. Moreover, those previous studies and the present data did not reveal significant differences in total plasma cholesterol concentrations between normal probands and type 1 and type 2 diabetic patients. Importantly, there was no correlation between the plasma HDL-cholesterol or HDL-apo A-I concentrations and the interaction of serum GPLD1 with micelle-like bAChE complexes (Figure 4) or serum GPLD1 activity/protein [30] (Figure 1): Taken together, the inverse regulation of the HDL parameters in type 1 (up compared to control) and type 2 (down compared to control) diabetic patients was faced by uniform upregulation of the serum GPLD1 parameters in diabetes type 1 and 2.

Remarkably, the same held true for the plasma HDL-cholesterol levels and the serum GPLD1 parameters for the six metabolically deranged rat groups. HDL-cholesterol levels were highest for lean ZFD (3.28 ± 0.36 mM, *p* ≤ 0.05 vs. lean Wistar) compared to lean ZF (2.45 ± 0.22 mM, *p* ≤ 0.05 vs. lean Wistar) and lean Wistar rats (1.59 ± 0.14 mM), and were significantly lower (*p* ≤ 0.05) for the obese (obese ZDF rats, 2.69 ± 0.20; obese ZF rats, 1.81 ± 0.14 mM; obese Wistar, 1.30 ± 0.11 mM) compared to the lean animals of the same genotype (see above). These findings are compatible with previously reported data [64,65,66,67]. Thus the plasma HDL-cholesterol levels of the six rat groups were not correlated to serum GPLD1 protein (Figure 1), activity towards bAChE complexes (Figure S1), and interaction with micelle-like bAChE complexes (Figure 2 and Figure 3). Each parameter increased with the ranking order lean < obese Wistar < lean ZF < obese ZF < lean ZDF < obese ZDF. In conclusion, the differences in serum GPLD1 protein, activity, and interaction with micelle-like bAChE complexes between the various human probands, as well as between the different rat groups, were not explained by different concentrations of HDL-cholesterol and apo A-I, but rather argued for differential interaction of (a) distinct serum factor(s) with GPLD1-micelle-like GPI-AP complexes.

### 3.3. Dependence of the Interaction between Serum Proteins, Not Identical with GPLD1, and the GPI Inositolglycan of Micelle-Like bAChE Complexes on Metabolic Derangement in Rats and Humans

**Figure 5:**
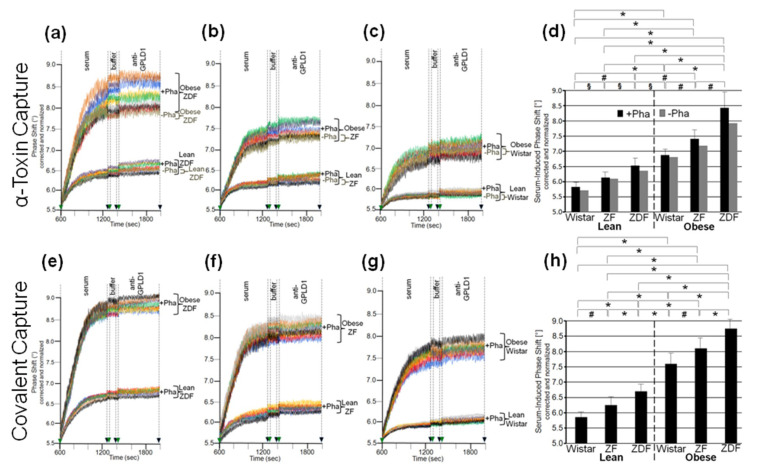
Measurement of the interaction of rat serum proteins not identical with GPLD1 with micelle-like bAChE complexes. (**a**–**c**) GPLD1-depleted sera were injected in the absence (dark brown curves only) or presence of Pha (0.5 mM, otherwise colored curves) into α-toxin-coated chips with captured micelle-like bAChE complexes. (**e**–**g**) GPLD1-depleted sera were injected in the presence of Pha (0.5 mM) into chips with covalently captured micelle-like bAChE complexes. After washing with buffer, anti-GPLD1 antibody was injected in the absence or presence of Pha in correspondence to the serum injection. (**a**–**c**,**e**–**g**) The experiment was performed and evaluated as described for Figure 4 with measurements shown only between 600 and 2000 s. The same chip was used for each rat group and the measurement repeated two times (different preparations of micelle-like bAChE complexes) with similar results (representatives shown). (**d**,**h**) The serum-induced phase shift (between 600 and 1300 s for serum injection and washing) determined in the presence of Pha (*n* = 8; triplicate measurements, black bars, means ± SD) or absence of Pha (*n* = 1; duplicate measurements, grey bars, shown only for α-toxin capture) is given. The comparison between the six rat groups is shown for the sera coinjected with Pha (black bars) only (* *p* ≤ 0.01, ^#^ *p* ≤ 0.02, ^§^ *p* ≤ 0.05).

The interaction between serum components other than GPLD1 and micelle-like bAChE complexes was investigated in greater detail. For this, rat serum was depleted of GPLD1 by immune adsorption and then analyzed for phase shift using chips for capture of complexes via α-toxin (Figure 5a–c) or covalent means (Figure 5e–g). Calculation of the means of the serum-induced phase shift in the presence of Pha revealed variance within the groups (eight individual rats) and significant differences between certain groups (Figure 5d,h, black bars). Obese ZDF rats exhibited the highest serum-induced phase shift (Figure 5a,e) and lean Wistar rats the lowest (Figure 5c,g). In the presence of Pha, the serum-induced phase shift significantly differed between lean or obese rats of different genotype, as well as between lean and obese rats of the same genotype (Figure 5d,h, black bars). The absence of Pha during serum injection did not affect the serum-induced phase shift (shown only for α-toxin capture; Figure 5a–d, grey bars). The apparent Ca^2+^-independence of and the impact of GPLD1 depletion on the serum-induced phase shift re-emphasized the interaction of serum components different from GPLD1 with the micelle-like complexes.

**Figure 6:**
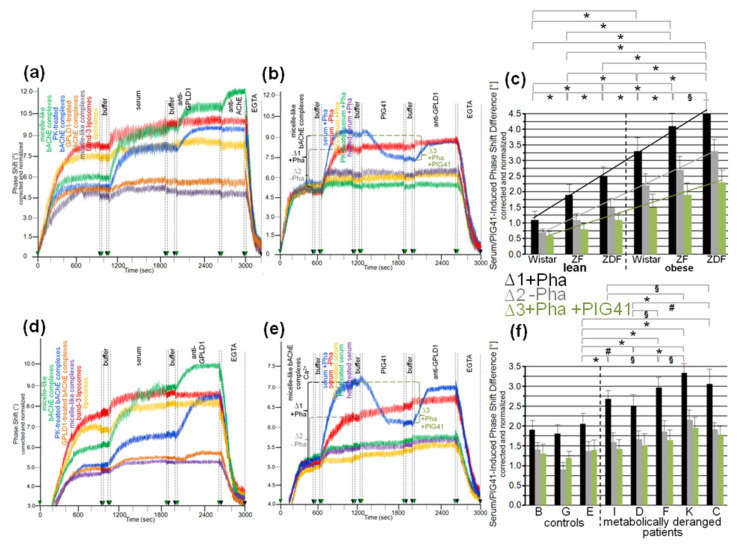
Structural requirements for the interaction of rat and human serum components with micelle-like bAChE complexes. Micelle-like complexes reconstituted in the absence (**a**,**d**, purple curves) or presence of bAChE at the “optimized” constituent ratio and then left untreated (**a**,**d**, green curves; **b**,**e**) or treated with proteinase K (PK; **a**,**d**, blue curves) or GPLD1 (**a**,**d**, brown curves) and liposomes reconstituted in the absence (**a**,**d**, orange curves) or presence of band-3 protein (**a**,**d**, red curves) were injected into uncoated chips in the presence of Ca^2+^ (5 mM). Following washing of the chips with buffer, serum from obese ZDF rats (**a,b**) or pooled serum from metabolically deranged patients (**d**,**e**) that had been left untreated (**a**,**d**; **b**,**e**, blue and red curves) or treated with proteinase K (PK; **b**,**e**, green curves) or heat (60 °C, 15 min; **b**,**e**, purple curves) or precipitated with PEG6000 (use of supernatant; **b**,**e**, orange curves) was injected in the presence of Pha (0.5 mM; **a**,**d**; **b**,**e**, blue, orange, green, purple curves) or its absence (**b**,**e**, red curves). After washing with buffer, PIG41 (30 µM) (**b**,**e** only), anti-GPLD1 antibody in the absence or presence of Pha in correspondence to serum injection, then anti-AChE antibody (**a** only) and finally EGTA were injected. Correction and normalization of the phase shift were performed as described for Figure 1. (**b**,**e**) The serum- and PIG41-induced phase shift difference in the presence (∆1, black lines and brackets) or absence of Pha (∆2, grey lines and brackets) or in the presence of Pha and then PIG41 (∆3, green lines and brackets) is indicated. (**c**,**f**) The experiment was performed with sera from the six rat groups (*n* = 8) and the individual human probands coinjected with Pha as described above for **b**,**e** (blue curves) and repeated three times (using the same chips and different preparations of micelle-like bAChE complexes) with similar results (16 and 8 regeneration cycles *per* chip, respectively). The serum- and PIG41-induced phase-shift difference (∆1, black bars and lines; ∆2, grey bars and lines; ∆3, green bars and lines) measured in quadruplicate is given as means ± SD. The comparisons between the rat groups (**c**) and human probands (**f**) are shown for the presence of Pha (∆1, black bars) only (* *p* ≤ 0.01, ^#^ *p* ≤ 0.02, ^§^ *p* ≤ 0.05). Linear regression analysis is indicated by the lines.

Next, the nature of the interaction between the micelle-like bAChE complexes and the rat or human serum components was characterized (Figure 6). Ionic capture of the differently pretreated micelle-like complexes and liposomes caused phase-shift increases, which were completely abrogated upon final regeneration of the chips with EGTA. Injection of serum from obese ZDF rats (Figure 6a,b) or metabolically deranged patients (pooled control sample, Figure 6d,e) and subsequently of anti-GPLD1 antibody induced similar phase-shift increases for micelle-like bAChE complexes that had been pretreated with proteinase K (Figure 6a,d, blue curves) or left untreated (green curves), but was without effect for complexes pretreated with GPLD1 (Figure 6a,d, brown curves) or lacking bAChE (Figure 6a,d, purple curves). The absence of bAChE was confirmed by subsequent injection of anti-AChE antibody, which did not cause any phase-shift increase (Figure 6a, brown and purple curves). Only moderate and no phase-shift increases upon serum and anti-GPLD1 antibody injections, respectively, were detectable for band-3 (Figure 6a,d, red curves) and “empty” liposomes (Figure 6a,d, orange curves). This was indicative of the presence of components in serum unspecifically interacting with liposomal phospholipids.

The phase-shift increases induced by rat (Figure 6b) or human serum (Figure 6e) in the absence of Pha (to prevent interaction with GPLD1) that had been treated with heat (purple curves), proteinase K (green curves), or PEG6000 (use of supernatant, orange curves) were considerably lower compared to untreated serum (Figure 6b,e, red curves). This argued for the proteinaceous nature of the rat and human serum component(s). Injection of PIG41 led to partial and no abrogation of the serum-induced phase-shift increases in the presence (Figure 6b,e, blue curves) and absence of Pha (red curves), respectively. The subsequent increase in the phase shift in response to anti-GPLD1 antibody with serum containing (Figure 6b,d, blue curves) but not lacking Pha (red curves) was compatible with GPLD1, being one of the serum proteins which interacts with micelle-like bAChE complexes. Apparently, this interaction, which can be detected only in the absence of Ca^2+^, was (partially) counteracted by PIG41. The interaction of the other serum proteins was not sensitive toward Ca^2+^.

Calculation of the means (eight individual rats *per* group) of the serum-/PIG41-induced phase-shift differences in the presence of Pha (total interacting proteins; Figure 6c,f, black bars), in the absence of Pha (interacting proteins without GPLD1; Figure 6c,f, grey bars) and in the presence of Pha and then PIG41 (interacting GPLD1; Figure 6c,f, green bars) revealed the highest and lowest values for obese ZDF and lean Wistar rats, respectively (Figure 6c) and metabolically deranged patients and controls, respectively (Figure 6f). The highest differentiation power was yielded with measurement of total interacting proteins (Figure 6c,f, black bars) compared to solely GPLD1 (green bars) and the others (grey bars). This resulted in significant differences between all rat groups (Figure 6c) as well as between the control “E” (displaying the highest serum-/PIG41-induced phase shift differences) and each of the metabolically deranged patients (Figure 6f). No significant differences were observed between the controls. In conclusion, distinct parts of the PIG moiety of the GPI anchor with high and low structural similarity to PIG41 seem to be involved in the interaction of micelle-like bAChE complexes with GPLD1 and the other rat or human serum proteins, respectively.

### 3.4. Impairment of Translocation of Full-Length GPI-APs from Micelle-Like Complexes to Rat Adipocytes by GPLD1 and Other Serum Proteins

The detection of the interaction of rat and human serum proteins, among them GPLD1, with full-length bAChE and its dependence on the metabolic derangement of the donors prompted the question about the (patho)physiological relevance of these findings. On basis of the previously reported translocation of full-length GPI-APs from detergent micelles, liposomes, and HDLs to vesicular and cellular membranes in vitro (see Discussion for details), the effect of serum proteins on the translocation of bAChE from micelle-like complexes to rat adipocytes during primary culture was investigated. The experimental protocol (see Materials and Methods for details) encompassed four steps: (i) translocation *per se*, (ii) recovery of the adipocytes with the translocated GPI-APs, (iii) spontaneous release of the translocated GPI-APs, and (iv) measurement of the amount of translocated, i.e., released, GPI-APs. In addition to bAChE, hCD73, CD55, and tissue-nonspecific alkaline phosphatase (aP) were used as model GPI-APs to study the potential impact of differences in the protein and GPI core glycan structures. hCD73, a dimer of 70-kDa subunits, exerts 5′-nucleotidase activity, thereby converting extracellular adenosine monophosphate to adenosine and playing important physiological roles, e.g., in vascular calcification [68,69]. CD55 or decay-accelerating factor (DAF), a 50 to 100 kDa monomer, binds to C4b and C3b complement factors at cell surfaces, thereby inhibiting early complement activation by accelerated degradation of C3 convertase and regulating both innate and adaptive immune responses [70,71]. aP, a 58-kDa monomer, converts pyrophosphate to inorganic phosphate, thereby fulfilling important physiological roles, e.g., in bone and tooth mineralization [72,73]. All these proteins represent ectoproteins that are anchored at the outer leaflet of mammalian plasma membranes by a covalently linked GPI moiety at its carboxy-terminus.

**Figure 7:**
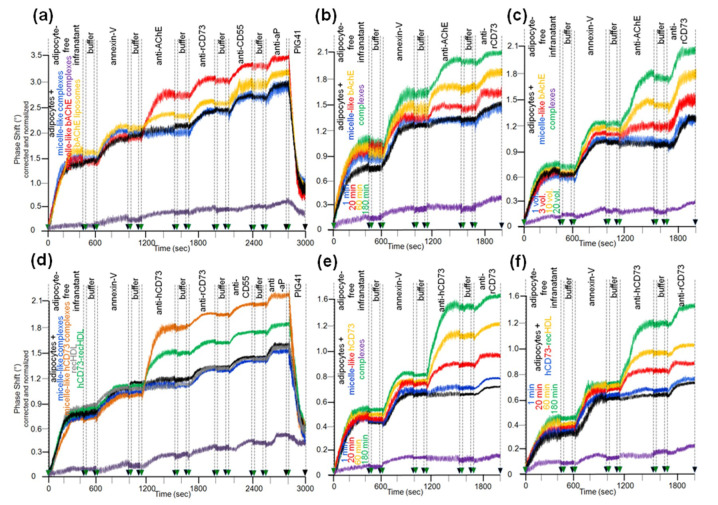
Translocation of bAChE and hCD73 from micelle-like complexes, liposomes, and HDLs to rat adipocytes. Isolated rat adipocytes were initially incubated (**a**,**c**,**d**, 180 min; **b**,**e**,**f**, 1–180 min) in the absence (black curves) or presence of liposomes (only **a**, orange curve), HDL (**d**,**f**, 10 volume equivalents) or micelle-like complexes (**a**,**b**,**d**,**e**, 20 volume equivalents; **c**, 1–20 volume equivalents) reconstituted without (**a**,**d**, blue and grey curves) or with bAChE (**a**, red, purple, and orange curves; **b**,**c**) or hCD73 (**d**, brown and green curves; **e**,**f**). The incubation mixtures were centrifuged through a cushion of dinonylphtalate. The adipocytes above the dinonylphtalate cushion were removed, washed two by flotation and then assayed for the amount of translocated bAChE (**a–c**) or hCD73 (**d**–**f**) by determination of their spontaneous release into micelle-like complexes upon subsequent incubation (37 °C) for 90 min (**a**–**f**) or 1 min (purple curves; **a**–**c**, only for micelle-like bAChE complexes; **d**,**e**, only for micelle-like hCD73 complexes; **f**, only for hCD73-recHDL). After centrifugation of the incubation mixtures through a cushion of dinonylphtalate, the adipocyte-free infranatants below the cushions were injected into α-toxin-coated chips. Following washing of the chips, the presence of GPI-APs, including the translocated bAChE and hCD73, was assayed by sequential injection of annexin-V (in the presence of Ca^2+^), anti-AChE (**a**–**c**), anti-rCD73 (**a**–**f**), and anti-hCD73 (**d**–**f**), as well as anti-CD55 and anti-aP (**a**,**d**) antibodies as indicated, with washings between each injection cycle. PIG41 was injected after the final antibody injection (**a**,**d**). Correction and normalization of the phase shift were performed as described for Figure 1.

In agreement with previous studies [28,30], rat adipocytes that had been incubated in the absence (Figure 7a–f, black curves) or presence of micelle-like complexes (Figure 7a–f, blue curves), liposomes (data not shown), and reconstituted HDLs (Figure 7d, grey curve, recHDL) released phospholipids, bAChE, CD73, CD55, and aP into micelle-like complexes. The efficacies were comparable as revealed by the very similar stepwise increases in the phase shift upon consecutive injection of the adipocyte-free infranatant, annexin-V, anti-AChE, anti-rCD73 (specifically recognizing rat CD73), anti-CD55, and anti-aP antibodies into α-toxin-coated chips (Figure 7a,d). This indicated that incubation with micelle-like complexes, liposomes, and recHDL lacking GPI-APs during step (i) does not provoke release of endogenous full-length GPI-APs, eventually due to induction of structural alterations of plasma membranes. The spontaneous release of the translocated full-length GPI-APs during step (iii) as measure for the initial translocation was demonstrated by almost complete abrogation of the phase shift upon reduction of the incubation period to 1 min (Figure 7a–f, purple curves). Furthermore, the missing phase shift in response to anti-AChE antibody recognizing both human and rat AChE was compatible with its low expression in adipocytes from rats, which apparently is in sharp contrast to the considerable expression of AChE reported for human adipocytes [74]. Strikingly, initial incubation with micelle-like bAChE complexes (Figure 7a, red curve), bAChE liposomes (Figure 7a, orange curve), micelle-like hCD73 complexes (Figure 7d, brown curve), and hCD73-recHDL (Figure 7d, green curve) harboring identical amounts of bAChE and hCD73, respectively, led to additional phase-shift increases in response to anti-AChE antibody and anti-hCD73 antibody (which specifically recognized human CD73). This argued for translocation of bAChE and hCD73 from the micelle-like complexes, liposomes, and HDLs to the rat adipocytes during the initial incubation. The efficacy was higher for the complexes compared to liposomes and HDL particles. Specific capture of the micelle-like complexes, liposomes, and recHDL harboring endogenous GPI-APs or exogenous bAChE/hCD73 by the chips was confirmed by elimination of the phase shift increases upon final injection of PIG41 (Figure 7a,d). The translocation of bAChE and hCD73 from micelle-like complexes (Figure 7b,e) or hCD73 from recHDL (Figure 7f) turned out to depend on the length of the initial incubation period (step i) with the adipocytes. It was clearly detectable already after 20 min and with three volume equivalents (shown only for bAChE complexes, Figure 7c) as demonstrated by the anti-AChE (Figure 7b,c) and anti-hCD73 (Figure 7e,f) antibody-induced phase-shift increases.

**Figure 8:**
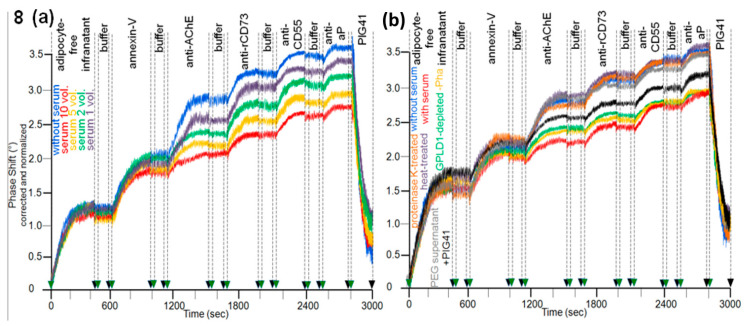
Effect of rat serum proteins on the translocation of bAChE from micelle-like complexes to rat adipocytes. The experiment was performed as described for Figure 7a with incubation (180 min) of isolated rat adipocytes with micelle-like bAChE complexes in the absence (**a**,**b**, blue curves) or presence of (**a**) serum from obese ZDF rats at increasing volumes (1–10 equivalents) together with Pha or (**b**) serum from obese ZDF rats (5 volume equivalents) that had been left untreated (red curve), or either subjected to heat (10 min, 60 °C; purple curve) or digested with proteinase K (brown curve), or precipitated with PEG6000 (use of supernatant; grey curve) or depleted of GPLD1 (green curve) together with Pha or untreated serum in the absence of Pha (orange curve) or untreated serum in the simultaneous presence of PIG41 and Pha (black curve). Correction and normalization of the phase shift were performed as described for Figure 1.

Having established the assay for the translocation of GPI-APs from micelle-like complexes to rat adipocytes, the effect of serum was studied. The presence of serum from obese ZDF rats during the initial incubation of micelle-like bAChE complexes with rat adipocytes led to a decline in translocation (Figure 8a, purple, green, orange, red curves) compared to the absence of serum (blue curve). bAChE together with (lyso)phospholipids, rCD73, CD55, and aP became released into micelle-like complexes of the adipocyte-free infranatant during the subsequent incubation (step iii) (Figure 8a). The specificity of the capture of the complexes by α-toxin-coated chips was confirmed by final injection of PIG41, which caused elimination of the phase-shift increases. Inhibition of the translocation was dependent on the amount of serum as demonstrated by more pronounced reductions of the antibody-induced phase-shift increases with increasing volume (Figure 8a). The intrinsic release of micelle-like complexes constituted by (lyso)phospholipids and endogenous GPI-APs from the rat adipocytes was not affected by serum. This was demonstrated by the similar phase-shift increases induced by the adipocyte-free infranatant and annexin-V in the absence (Figure 8a, blue curve) and presence of serum (other colors). Thus, the intrinsic release of GPI-APs during step (iii) can be used as measure for the translocated bAChE.

The nature of the serum components responsible for inhibition of the translocation of bAChE from micelle-like complexes to rat adipocytes was studied with serum from obese ZDF rats (Figure 8b). This had been treated with heat (Figure 8b, purple curve), proteinase K (brown curve), PEG6000 (grey curve; supernatant used only), or depleted of GPLD1 (green curve) or incubated in the absence of Pha (orange curve) or presence of PIG41 (black curve). Each treatment diminished the potency of serum to impair translocation compared to untreated one (Figure 8b, red curve) by variable degree to up to the translocation rate as measured in the absence of serum (Figure 8b, blue curve). The latter was achieved by complete deproteinization through heat, proteinase K, or precipitation. Together, these data argued that serum proteins, among them GPLD1, interfere with translocation of bAChE from micelle-like complexes to rat adipocytes. The partial abrogation of the serum-induced translocation inhibition by PIG41 (Figure 8b, black curve) suggested that some of those serum proteins interfere with GPI-AP translocation through interaction with the PIG portion of the GPI anchor. Final proof of the direct interaction of GPI anchor with serum proteins will require the use of GPI-APs that exhibit the complete PIG core glycan but different protein moieties, including truncated ones.

### 3.5. Dependence of the Impairment of GPI-AP Translocation by Serum Proteins on the Metabolic Derangement in Rats and Humans

**Figure 9:**
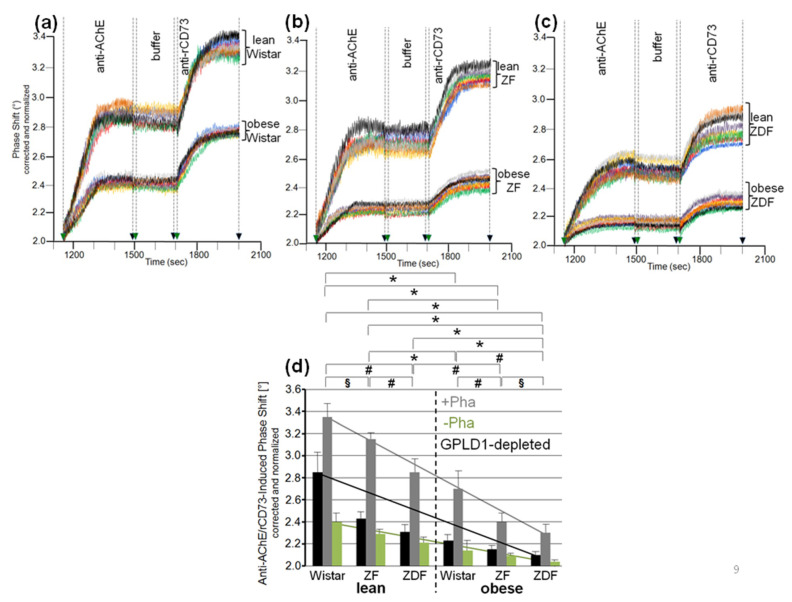
Differential inhibition of translocation of bAChE from micelle-like complexes to rat adipocytes by serum from metabolically deranged rats. The experiment was performed as described for Figure 8 with initial incubation (180 min) of isolated rat adipocytes with micelle-like bAChE complexes (20 volume equivalents) and serum (5 volume equivalents) that had been left untreated in the presence of Pha (**a**–**d**) or its absence (only **d**), or that had been depleted of GPLD1 in the presence of Pha (only **d**). (**a**–**c**) Phase shift is shown only for the injection of anti-AChE and anti-rCD73 antibodies (between 1150 and 2000 s). Each comparison was performed with the same chip and repeated three times (different preparations of micelle-like bAChE complexes) with similar results (representatives shown). (**d**) The anti-AChE and rCD73 antibody-induced phase shift between 1150 and 2000 s (*n* = 8; triplicate measurements, means ± SD) obtained upon incubation of the adipocytes with micelle-like bAChE complexes and untreated serum in the absence (green bars) or presence of Pha (grey bars), or serum depleted of GPLD1 in the presence of Pha (black bars) and following transfer of bAChE as the amount of bAChE finally released is given. The comparison between the six rat groups is shown for sera treated with Pha (grey bars) only (* *p* ≤ 0.01, ^#^ *p* ≤ 0.02, ^§^ *p* ≤ 0.05). Each comparison was performed with the same chip and repeated two times (different preparations of micelle-like bAChE complexes) with similar results. Linear regression analysis is indicated by the lines.

Analysis of the eight individual sera from each of the six rat groups for impairment of translocation of bAChE from micelle-like complexes to rat adipocytes in the presence of Pha (for inclusion of GPLD1) revealed variance within the groups and significant differences between them (Figure 9a–c). Serum from obese ZDF rats exhibited the highest inhibition of translocation (Figure 9c), followed by obese ZF (Figure 9b), then obese Wistar and finally lean Wistar rats with the lowest inhibition (Figure 9a). In the presence of Pha, the means (of the groups) for the anti-AChE/rCD73 antibody-induced phase shift as measured for the bAChE translocation left revealed significant differences between certain rat groups, such as between lean or obese rats of different genotypes, as well as between lean and obese rats of the same genotype (Figure 9d, grey bars). Omission of Pha (Figure 9d, green bars) or depletion of GPLD1 (black bars) (for exclusion of GPLD1) did not alter the ranking order for translocation inhibition by the sera, albeit the differences between the groups did not reach significance.

**Figure 10:**
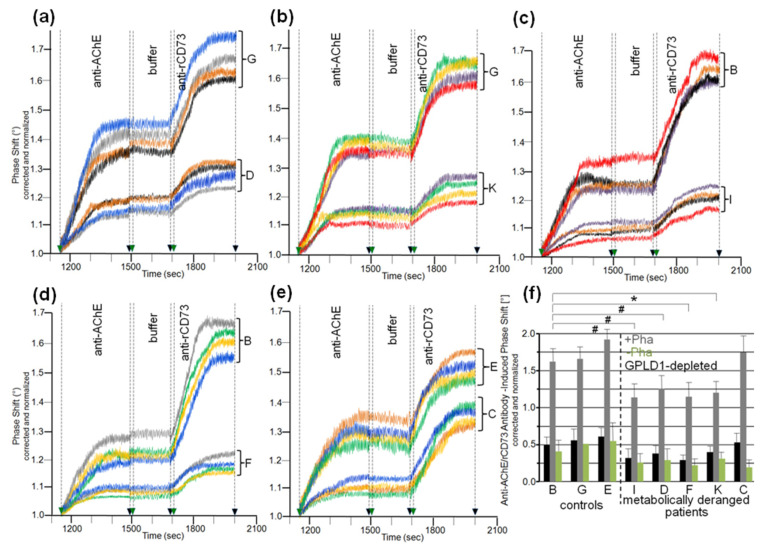
Differential inhibition of translocation of bAChE from micelle-like complexes to rat adipocytes by human serum. The experiment was performed as described for Figure 9 with initial incubation (180 min) of isolated rat adipocytes in the presence of micelle-like bAChE complexes (15 volume equivalents) and serum (10 volume equivalents) from controls or metabolically deranged patients (**a**–**e**) that had been left untreated in the presence of Pha (**a**–**f**) or its absence (only **f**), or that had been depleted of GPLD1 in the presence of Pha (only **f**). The limited number of probands prevented the clustering of age- and gender-matched cases and control groups. Therefore, samples “G” and “B” were used twice as controls for comparison with samples “D” and “K” (**a**,**b**) or “I” and “F” (**c**,**d**), respectively, and sample “E” was used once as control for sample “C” (**e**) during the same run. (**a**–**e**) Phase shift was measured in quadruplicate as indicated by the different colors of the curves with different incubations of the adipocytes following transfer of bAChE for its assaying as the amount subsequently released. Four independent measurements were performed *per* proband (distinct chips with different micelle-like bAChE complexes) indicated by the different colors of the curves. (**f**) The anti-AChE and rCD73 antibody-induced phase shift is given as means ± SD (*n* = 4) for incubation of the adipocytes with micelle-like bAChE complexes and serum that had been left untreated (green bars) or treated with Pha (grey bars), or depleted of GPLD1 and treated with Pha (black bars). The comparison between the probands is shown for sera treated with Pha (grey bars) only (* *p* ≤ 0.01, ^#^ *p* ≤ 0.02).

Analysis of serum samples from the eight human probands (Table 2) for inhibition of translocation in the presence of Pha revealed differences in the anti-AChE/rCD73 antibody-induced phase shift (Figure 10a–e). These were nonsignificant between the three controls, as well as between the five metabolically deranged patients, but significant between controls and patients (Figure 10f, grey bars). Sera from the patients exerted a lower anti-AChE/rCD73-induced antibody-induced phase shift, i.e., higher inhibition of bAChE translocation, compared to control “B”, which displayed the lowest phase shift among the controls. Omission of Pha (Figure 10f, green bars), as well as use of GPLD1-depleted serum (Figure 10f, black bars), left (nonsignificant) trends between the controls and the metabolically deranged patients. As was observed with rats (Figure 9), the inhibitory potency of GPLD1-depleted human serum proteins was lower than that of total serum proteins. This argued for a concerted action of certain serum proteins and GPLD1 in the inhibition of translocation of GPI-APs to the surface of acceptor cells. This is presumably based on the interaction of those serum proteins with GPI anchors, with different proteins possibly involving distinct parts of the anchor.

### 3.6. Lysis of Rat Adipocytes by Micelle-Like bAChE Complexes and Its Prevention by Serum Proteins from Metabolically Deranged Rats

**Figure 11:**
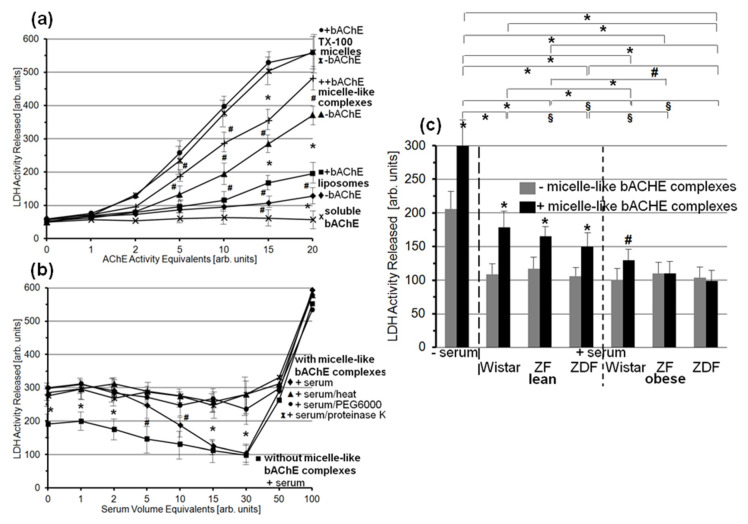
Effect of micelle-like bAChE complexes and serum on LDH release from rat adipocytes. (**a**) Isolated rat adipocytes were incubated (180 min, 37 °C) with micelle-like complexes that had been reconstituted in the absence (▲) or presence of bAChE (+), or with TX-100 micelles that had been reconstituted in the absence (Ж) or presence of bAChE (•), or with liposomes that had been reconstituted in the absence (♦) or presence of bAChE (■), or with soluble bAChE that had been prepared by GPLD1 treatment of purified bAChE and subsequent TX-114 partitioning (use of detergent-depleted phase, see Materials and Methods; **x**) at increasing AChE activity equivalents (micelle-like complexes, TX-100 micelles, and liposomes were adjusted to the same AChE volume activity). (**b**) Isolated rat adipocytes were incubated (180 min, 37 °C) without (■) or with micelle-like bAChE complexes (10 activity equivalents; ♦, •, ▲, Ж) in the absence or presence of serum (at increasing volume equivalents) from obese ZDF rats that had been subjected to heat (10 min, 60 °C; ▲) or digested with proteinase K (Ж), or precipitated with PEG6000 (use of supernatant; •), or left untreated (♦, ■). LDH activity measured for three incubations each with enzymic assays in quadruplicate is given as means ± SD (* *p* < 0.01, ^#^ *p* < 0.02). (**c**) Isolated rat adipocytes were incubated (180 min, 37 °C) without (grey bars) or with micelle-like bAChE complexes (10 AChE activity equivalents; black bars) in the absence or presence of rat serum (30 volume equivalents). LDH activity measured for four independent incubations each with enzymic assays in triplicate is given as means ± SD for the presence (black bars) and absence (grey bars) of micelle-like bAChE complexes and for each serum or no serum (* *p* ≤ 0.01, ^#^ *p* ≤ 0.02). The comparison of the LDH activity released between the six rat groups is shown for the incubations with micelle-like bAChE complexes (black bars) only (* *p* ≤ 0.01, ^#^ *p* ≤ 0.02, ^§^ *p* ≤ 0.05).

The finding of translocation of bAChE from micelle-like complexes to rat adipocytes raised the possibility of impairment of the integrity of adipocyte plasma membranes in the course of intercalation of the bAChE GPI anchor between the phospholipids of the outer leaflet. Consequently, the effect of micelle-like bAChE complexes on lysis of cultured adipocytes was assayed using the release of cytoplasmic LDH as the criterion. In fact, incubation of primary rat adipocytes with micelle-like bAChE complexes led to elevated LDH activity in the culture medium (Figure 11a, + curve). This was positively correlated to the amount of complexes used (corresponding to AChE activity equivalents). Complexes lacking bAChE (Figure 11a, ▲ curve) as well as liposomes containing (■ curve) or lacking (♦ curve) bAChE exerted significantly lower LDH activity increases. In contrast, TX-100 micelles containing (Figure 11a, • curve) or lacking (Ж curve) bAChE led to the highest LDH activity released. The increases in complex-, liposome- and micelle-induced LDH release in the presence vs. absence of bAChE and the missing effect of soluble bAChE lacking the GPI anchor (Figure 11a, x curve) on LDH release hinted at the bAChE GPI anchor as the mediator of adipocyte lysis in the course of translocation to the cell surface via micelle-like or vesicular structures.

The presence of serum (from obese ZDF rats) during incubation of rat adipocytes with micelle-like bAChE complexes (Figure 11b) led to reduction in LDH release depending on the amount of serum (♦ curve) to up to the value for incubation in the absence of micelle-like bAChE complexes (thereby corresponding to intrinsic adipocyte lysis; ■ curve). At amounts exceeding this threshold, the serum apparently exerted detrimental effects on adipocyte viability, which caused drastic increases in LDH release irrespective of the presence (Figure 11b, ♦ curve) or absence (■ curve) of micelle-like complexes. The proteinaceous nature of the serum component(s) responsible for prevention of bAChE complex-induced adipocyte lysis was demonstrated by the use of heated (Figure 11b, ▲), proteinase K-digested (x), and PEG6000-precipitated (•; supernatant used only) serum. The resulting LDH release did not differ from that in the absence of serum, but was significantly higher compared to untreated serum with increasing (10–30) volume equivalents (Figure 11b, ♦ curve).

Finally, sera from the six rat groups were assayed for putative differences in the inhibition of LDH release upon incubation of rat adipocytes with micelle-like bAChE complexes (Figure 11c). For this, serum from obese ZDF rats at 30 volume equivalents was used, which mediated complete prevention of complex-induced lysis. Each serum led to significant reduction in LDH release in the presence (Figure 11c, black bars) as well as absence (grey bars) of micelle-like bAChE complexes. The effect on complex-independent lysis did not vary with the source of the serum. This was compatible with an “overall” protection of adipocytes toward lysis by serum components to up to a certain concentration (Figure 11b). In contrast, the resistance toward bAChE complex-induced lysis was most pronounced with serum from obese ZDF and ZF rats, followed by obese Wistar rats, and lowest with lean Wistar rats (Figure 11c). The means of the complex-induced LDH release revealed significant differences between certain rat groups, among them between lean and obese rats of the same genotype, lean or obese Wistar and ZDF rats, lean ZDF and obese ZF rats, as well as lean ZF and obese Wistar rats. Together, the data suggested that rat serum contains proteins that protect rat adipocytes against lysis provoked by micelle-like GPI-AP complexes. This protecting activity was dependent on the metabolic derangement of the rats.

## 4. Discussion

### 4.1. GPLD1 as Metabolic Stress-Induced GPI-Interacting and Degrading Protein

Soon after identification of GPLD1 in rodent and human serum and liver, the regulation of its amount and activity by various metabolism-related factors has attracted much interest. Typically, upregulation of GPLD1 hepatic protein expression and serum level in the diabetic state was found, such as for spontaneously type I diabetic mice [75], NOD and low-dose Streptozotocin-induced diabetic CD-1 mice [54], db/db mice [76], Streptozotocin-induced diabetic mice [76] and rats [77,78], and mice fed with high-fat and high-sucrose diet [76]; and for patients with non-alcoholic steatohepatitis [79], latent autoimmune diabetes in adults [80], type 1 diabetes [80], insulin-resistance [81], and type 2 diabetes [82]. Downregulation of GPLD1 hepatic expression was reported for obese nondiabetic women on a low-fat diet with re-gain in insulin sensitivity [83]. Complete loss of GPLD1 protein in serum upon deletion of the *GPLD1* gene in mice was shown to result in improvement of glucose tolerance and hepatic steatosis under a high-fat and high-sucrose diet [84].

However, the correlation between GPLD1 hepatic gene expression or serum level and metabolic derangement turned out to be rather weak. This presumably has prevented use of GPLD1 protein as biomarker for diabetes or obesity so far. This holds even more true for GPLD1 activity, which was only very moderately increased in serum from diabetic and old rats and mice, as well as from type 1 diabetic patients [77,84,85,86]. Moreover, two studies failed to confirm correlation between GPLD1 protein and activity in serum as well as liver and the hyperinsulinemic/hyperglycemic state at all. Rather, they reported that GPLD1 overexpression in liver of mice with resulting sevenfold increase in serum GPLD1 level led to improvement of oral glucose tolerance [87] and reduction in serum triglyceride catabolism [88].

Importantly, the finding of inactivity of GPLD1 toward GPI-APs embedded in intact (cellular, vesicular, liposomal) membranes in most [36,37,38,89,90,91] but not all [92,93] studies made less likely the possibility that the site of GPLD1 action in vivo is at the interface between blood/tissue cell surfaces and the blood compartment. Rather, hepatic clearance, i.e., endocytotic uptake of GPI-APs by liver cells and subsequent lipolytic cleavage in their endolysosomal compartment, fostered by the bile salt-induced detergent-like environment, has been suggested as the physiological, i.e., intracellular, role of GPLD1 [94,95,96,97]. Nevertheless, for methodological limitations in most studies reported so far, GPLD1 activity measurement has been performed with serum rather than liver samples in combination with GPI-APs presented in detergent micelles.

The recent observations that (i) bAChE reconstituted into micelle-like complexes together with (lyso)phospholipids and cholesterol acts as more efficient substrate for serum GPLD1 than mixed GPI-AP detergent micelles or GPI-AP liposomes [28] and that (ii) GPI-APs are present in complexes of similar structure in rat and human serum [30] renewed the speculation about the blood compartment as the physiological site of degradation of GPI-APs. Accordingly, GPLD1 action under physiological conditions would necessitate the coexpression or coinsertion of GPLD1 and GPI-APs in the same exofacial phospholipid leaflet of plasma membranes or of plasma lipoproteins or micelle-like complexes, i.e., of those structures which have been demonstrated to harbor GPI-APs so far [18,19,20,21,22,23,24,25,26,27,30,56].

Using a combination of affinity purification and dot blotting, low amounts of CD73, CD55, and AChE were detected in HDLs from human plasma samples (Table 3a). Remarkably, HDLs from type 1 and type 2 diabetic patients tended to harbor higher concentrations of these GPI-APs compared to control probands. Upregulation of CD73 and CD55 expression in HDLs reached significance for diabetic patients vs. controls rather than total cholesterol, HDL-cholesterol, and apo A-I levels (Table 3b). The apparent accumulation of certain GPI-APs at HDL in plasma of type 1 and 2 diabetic patients vs. controls is in contrast to the reduced steady-state concentration of micelle-like GPI-AP complexes, as has been demonstrated previously for the same samples [28]. The chip-based analysis used specifically monitored micelle-like GPI-AP complexes rather than HDLs. This upregulation of HDL expression of GPI-APs may be explained by overriding the degradation capacity of GPLD1 bound to HDLs in the course of massive appearance in the blood and insertion into HDLs of released GPI-APs in response to metabolic derangement. Alternatively, HDL may provide an unfavorable milieu for GPLD1 for cleavage of the inserted GPI-APs in comparison to their presentation in micelle-like complexes. In the present study, apo A-I, which is known to stimulate HDL-associated GPLD1 activity in vitro [54,55], was identified in the plasma HDLs at typical amounts [60,61] and did not differ significantly between diabetic patients and controls (Table 3b). Thus, changes in apo A-I expression do not account for downregulated degradation and consequently elevated levels of CD73 and CD55 in plasma of the diabetic vs. control probands. However, it remains to be clarified whether apo A-I and GPI-APs reside within the same HDL particles. The ratio between HDLs expressing either apo A-I or CD73/CD55 and HDLs expressing both of them may determine upregulation of GPI-AP expression in plasma HDLs of diabetic patients.

Future research is required to delineate the (major and minor) pathways taken by full-length GPI-APs upon their (metabolic stress-induced) release from the surface of tissue and blood cells into the circulation, as well as the sites of their cleavage by GPLD1, i.e., micelle-like complexes vs. HDLs.

The present findings demonstrate dependence of the GPLD1 activity toward micelle-like bAChE complexes (Figure S1) and, less pronouncedly, GPLD1 protein (Figure 1) in serum, as determined by chip-based sensing, on the glycemic and insulinemic state of high-fat diet-fed and genetic rat models of diabetes and obesity with the ranking order obese ZDF > obese ZF > obese Wistar > lean ZDF > lean ZF > lean Wistar. Moreover, the data argue for the (patho)physiological relevance of the lipolytic cleavage of GPI-APs, arranged together with (lyso)phospholipids and cholesterol in micelle-like complexes, by GPLD1 in the blood compartment. This was reinforced by determination of the interaction between serum GPLD1 of the six rat groups and bAChE. The dependence of the amount of bAChE-interacting GPLD1 on the hyperglycemic hyperinsulinemic state (Figure 1) was found to be more pronounced than that of the GPLD1 activity (Figure S1).

The apparently more pronounced dependence of the interaction with bAChE of serum GPLD1 compared to its activity and even more protein on the metabolic state of the six rat groups may be explained by an activation factor in serum. Its binding via hydrophobic interactions to and activation of GPLD1 have been postulated previously on the basis of indirect experimental findings [30]. Over the past three decades, multiple modes of regulation of GPI-PLD activity, among them posttranslational modifications, such as proteolytic processing [98] and threonine phosphorylation [99], as well as stimulation and inhibition by naturally occurring amphiphiles [90,100,101,102], have been suggested.

### 4.2. Metabolic Stress-Induced GPI-Interacting Proteins in Serum

Interestingly, even upon omission of Pha with the resulting abrogation of the interaction between bAChE and GPLD1 or upon depletion of GPLD1 from serum, dependence of the amount of bAChE-interacting serum proteins on the metabolic derangement was preserved for the six rat groups (Figure 2, Figure 3, Figure 5 and Figure 6) and the eight human probands (Figure 4). It was, however, less pronounced compared to that measured in the presence of Pha and GPLD1 (Figure 6c,f, black and grey bars). These findings provided a first hint of the presence of components in rat and human serum that specifically interact with GPI-APs in micelle-like complexes and are not identical to GPLD1.

The measurement of the interaction of serum proteins with micelle-like bAChE complexes enabled the discrimination of healthy controls and metabolically deranged patients (Figure 4c,d). The significant differences herein within the latter (but not the former), who displayed considerable variability with regard to body weight and hyperglycemic/hyperinsulinemic state (Table 2), opens the possibility that a strong positive correlation between this interaction and the metabolic phenotype will be established in future studies with larger numbers of probands. For this, the use of a mutant version of bAChE lacking large portions of its polypeptide moiety but retaining its complete PIG moiety may present an advantage. Thereby, unspecific interactions not relying on the GPI anchor but putatively masking any correlation will be excluded.

Pretreatment of the micelle-like bAChE complexes with proteinase K or GPLD1 and the use of complexes lacking bAChE demonstrated that the GPI anchor is critical for the interaction of rat and human serum components (Figure 6a,d). For both rats and humans, the nature of the serum factors turned out to be proteinaceous (Figure 6b,e). Importantly, the epitopes of the GPI anchor that are recognized by GPLD1 and the other serum protein(s) apparently differ. Only the interaction of the latter with the micelle-like bAChE complexes was competitively blocked by PIG41, which resembles the PIG portion of GPI anchors (Figure 6b,e). In agreement, strict dependence was observed for the PIG41-sensitive interaction of total serum proteins with the complexes on the hyperglycemic/hyperinsulinemic state of the rats and human probands (Figure 6c,f, green bars). Together, the data argue for interaction of a subset of serum proteins, among them GPLD1, with full-length GPI-APs in micelle-like complexes in rats and humans that is controlled by their metabolic state. To evaluate the value of the observed dependence of serum GPLD1 amount, activity, and interaction with GPI-APs on the blood glucose and plasma insulin levels for the prediction and diagnosis of metabolic diseases, correlation analyses with determination of putatively positive correlation coefficients will be performed for larger numbers of prediabetic, diabetic, overweight, and obese rats, as well as humans, in the future.

### 4.3. Prevention of Metabolic Stress-Induced Translocation of GPI-APs to and Lysis of Acceptor Cells by GPLD1 and Other Serum Proteins

The above finding that GPLD1 and other serum proteins interact with micelle-like bAChE complexes in vitro opens the possibility that they interact with full-length GPI-APs in the circulation upon their release from the surface of blood or tissue cells and presentation in complex with (lyso)phospholipids and cholesterol. It is tempting to speculate that the potentially deleterious effects of full-length GPI-APs in the circulation upon their contact with blood or tissue cells are prevented by the interaction with certain serum proteins and GPLD1, prior to or alternatively to or in combination with lipolytic removal of their GPI anchor. In this regard, previous reports about the intermembrane transfer of full-length GPI-APs, independently published by two groups even before the first identification of GPI anchors, are of considerable relevance. During the study of phospholipid exchange between liposomes and cells, Bouma and coworkers found that certain membrane proteins, among them AChE, become translocated from erythrocytes to liposomes [31]. Medof and coworkers showed that the detergent-solubilized purified human erythrocyte membrane proteins, DAF or CD55, firmly associate with sheep erythrocytes upon incubation [103]. Importantly, erythrocyte-associated DAF maintained the biological activity of its detergent-solubilized version. Subsequent research by other groups confirmed the ability of GPI-APs to be translocated from detergent micelles to liposomal and cellular membranes or transferred between cellular membranes under preservation of their functionality [104,105,106,107,108]; for a review, see [109].

In agreement, using the novel chip-based assay design, the translocation of bAChE in concert with other GPI-APs was demonstrated in the present study and found to occur with higher efficacy upon reconstitution of bAChE into micelle-like complexes compared to liposomes (Figure 7). Strikingly, the presence of serum during the incubation caused inhibition of transfer (Figure 8a), which was not observed upon denaturation, digestion, or removal of protein and diminished by inhibition or removal of GPLD1 or by presence of PIG41 (Figure 8b). These data argue that full-length bAChE is prone to translocation from micelle-like complexes to rat adipocytes in vitro, and that certain serum proteins block the translocation in the course of interaction with the PIG portion of its GPI anchor. A (patho)physiological role for the prevention of translocation of full-length GPI-APs by (both total and GPLD1-depleted) serum proteins was suggested by dependence of prevention on the metabolic derangement in rats (Figure 9). In addition, measurement of this inhibitory potency of (total and GPLD1-depleted) serum proteins enabled the discrimination of healthy controls and metabolically deranged patients (Figure 10).

These findings are compatible with previous reports about inhibition of translocation of GPI-APs by lipid- and fatty-acid-binding proteins, such as albumin and lipoproteins [103,110]. Importantly, those experimental protocols enabled the translocation of purified GPI-APs to plasma membranes from the aqueous milieu lacking any [111,112] or containing only very low concentrations of detergent [110,113]. Thus, it is conceivable that the translocation of full-length GPI-APs from the blood compartment to tissue cells involves the dissociation of the GPI fatty acyl chains from the micelle-like complexes. The latter may shield those acyl chains from the aqueous milieu of the blood upon their contact with and insertion into plasma membranes. Interestingly, Suzuki and Okumura previously reported that GPI-APs fail to become spontaneously translocated from erythrocytes to liposomes as well as transferred between mammalian cells in the absence of exogenous proteins [35,114]. Thus, the translocation of GPI-APs from micelle-like complexes to cell surfaces as monitored here in vitro and their intercellular transfer in vivo may depend on the activity of hitherto unknown proteinaceous catalysts. It is conceivable that certain serum proteins, including GPLD1, act as inhibitors of those catalysts through interaction with full-length GPI-APs. In conclusion, chip-based sensing of GPI-APs has already revealed novel insights into the molecular mechanism of GPI-AP transfer between cells, and may facilitate future studies about its (patho)physiological roles. Apparently, this has been hampered during the past decades by the experimental expenditure for the detection of full-length GPI-APs.

Considering the amphiphilic nature of full-length GPI-APs, their physical contact with blood and tissue cells could exert direct damage on them, at least at high local concentrations. In fact, in contrast to soluble anchorless bAChE, full-length bAChE in micelle-like complexes caused lysis of rat adipocytes in vitro in a concentration-dependent fashion (Figure 11a). The possibility of prevention of complex-induced adipocyte lysis by serum proteins was studied. In fact, the prevention was found to hold true with native, but not denatured, digested and protein-depleted serum (Figure 11b). Strikingly, the inhibitory potency of serum proteins on complex-induced adipocyte lysis (Figure 11c) paralleled that of complex-mediated GPI-AP translocation (Figure 9). Both of them increased with metabolic derangement of the rats. This argues for mechanistic linkage between GPI-AP translocation to and complex-induced lysis of blood/tissue cells, as well as for prevention of translocation and lysis by certain serum proteins.

Together, the experimental evidence available hints to the following physiological roles of serum proteins which interact with full-length GPI-APs in micelle-like complexes: (i) blockade of translocation of GPI-APs to the surface of blood and tissue cells, which typically do not produce them and which may suffer from functional surface expression of the binding, enzymic, or signaling activities exerted by the translocated GPI-APs; and (ii) prevention of lysis of blood and tissue cells upon contact with micelle-like GPI-AP complexes at (too) high concentrations.

### 4.4. Conclusions

**Figure 12:**
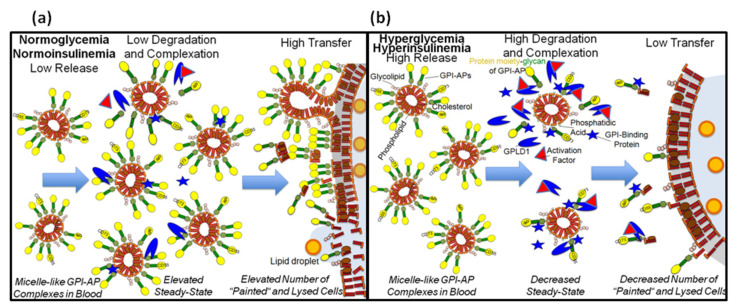
Model for the path of full-length GPI-APs in micelle-like complexes in mammalian blood. (**a**) Full-length GPI-APs from the surface of relevant donor blood/tissue cells, such as adipocytes, are released into micelle-like complexes in the blood in the normoglycemic normoinsulinemic state at low rate. This is paralleled by the degradation of the GPI-APs through lipolytic cleavage of their GPI anchor by GPLD1 at a low rate, leading to accumulation of the complexes with time. The resulting steady-state concentration of full-length GPI-APs in micelle-like complexes is faced with a considerably low amount of GPI-interacting proteins, among them GPLD1, which associate with the GPI anchor via the glycan core. Together, these conditions enable the efficient translocation of full-length GPI-APs from the micelle-like complexes or fragments generated thereof into the outer leaflet of plasma membranes of relevant acceptor blood/tissue cells, such as adipocytes. Overall, this intercellular transfer leads to considerable increase in the number of “painted” cells, i.e., cells expressing exogenous GPI-APs at their surface. Eventually, as a consequence of excessive transfer, the “painted” cells may undergo lysis in the course of disintegration of their plasma membranes by the translocated amphiphilic full-length GPI-APs. (**b**) The upregulated release of full-length GPI-APs into micelle-like complexes in the blood in the hyperglycemic hyperinsulinemic state becomes counteracted by their degradation through GPLD1 at an elevated rate. The resulting steady-state concentration of full-length GPI-APs in the micelle-like complexes is lower compared to that of the normoglycemic normoinsulinemic state. This, in combination with efficient interaction of the full-length GPI-APs with GPI-interacting proteins, prevents their translocation to the surface of acceptor blood/tissue cells, leading to their diminished “painting” and “lysis” and impaired intercellular transfer of GPI-APs.

The data presented in combination with previous reports [28,29,30] can be reconciled in a model for the processing of full-length GPI-APs following their functional cell-surface expression. It considers the observed interrelationship between upregulation of their release into as well as removal from blood, and increases in blood glucose and/or insulin (Figure 12). The mechanistic linkage between release of GPI-APs and glucose/insulin levels remains to be clarified, but may rely on certain biophysical characteristics of the plasma membranes of donor blood and tissue cells, such as elasticity, viscosity, and stiffness. The coupling between removal of GPI-APs and metabolic blood parameters may rely on their arrangement in micelle-like complexes. These may induce upregulation of the expression of GPLD1, activation factor, and those serum proteins capable of interacting with micelle-like GPI-AP complexes by unknown molecular mechanism(s). In addition, the concerted action of those serum proteins may prevent the deleterious effect of full-length GPI-APs upon contacting blood and tissue cells. It remains open whether GPI-AP translocation and GPI-AP-induced lysis are independent events that affect distinct cell populations of varying susceptibility toward the complexes. Alternatively, there may be a causal relationship with the translocated GPI-APs destabilizing the plasma membrane outer leaflet.

In the future it will be investigated whether monitoring of the processing of full-length GPI-APs in the circulation enables (i) the prediction of the development of metabolic diseases prior to manifestation in elevated blood glucose and plasma insulin levels, and (ii) their stratification on basis of the identification of those tissues and organs, which release full-length GPI-APs or to which these become translocated as cause for or consequence of pathogenic events. In achieving this goal, the major advantage of SAW sensing of biomolecular interactions would have been reinforced: The accurate and sensitive detection of the total of full-length GPI-APs and associated proteins in serum that fails to be managed by conventional (co)immune precipitation/immune blotting due to lack of appropriate antibodies. Furthermore, it will be feasible to combine the chip-based sensing of GPLD1 and other GPI-AP-interacting serum proteins in vitro with that of full-length GPI-APs present in complex with (lyso)phospholipids and cholesterol in serum in vivo into an integrated homogenous assay platform. For this, the chip surface has to be coated with a nonsaturating amount of micelle-like bAChE complexes via ionic capture to enable the subsequent capture of serum complexes by α-toxin. At variance, in the present study the capture capacity of the chips was saturated with an excess of micelle-like bAChE complexes in order to ensure comparable capture efficacy of different chips and to avoid interference with eventual and unwanted capture of serum complexes. This multiplex assay platform may broaden the spectrum of biomedical applications that have been envisaged so far for GPI-APs [115].

## Figures and Tables

**Table 1:** Characteristics of the rats. Mean values of weight, fasting blood glucose, and fasting plasma insulin for each rat group (of given genotype and feeding) are given in bold.

Geno-Type	Feeding	Weight [g]	Age [Weeks]	Fasting Blood Glucose [mM]	Fasting Plasma Insulin [µg/L]	Pheno-Type
Wistar	lean	309.7 356.4 336.8 321.5 360.7 376.3 331.8 358.9 **344.0**	10 10 10 10 10 10 10 10	5.49 5.87 5.94 6.23 6.10 6.81 7.25 7.87 **6.44**	0.56 0.48 0.59 0.65 0.74 0.86 1.46 1.69 **0.88**	normo-glycemic normo-insulinemic
	obese	509.6 469.3 496.1 481.4 561.0 482.1 523.9 580.4 **512.0**	10 10 10 10 10 10 10 10	6.36 6.08 6.75 6.97 6.44 6.82 7.45 7.90 **6.85**	0.89 1.74 2.73 1.97 1.63 2.94 1.83 2.19 **1.99**	normo-glycemic mildly hyper-insulinemic
ZF	lean	435.3 496.5 473.2 450.8 505.2 479.3 499.5 421.7 **470.2**	40 40 40 40 40 40 40 40	6.27 5.75 5.42 5.99 6.34 5.81 5.12 5.93 **5.83**	1.24 0.87 0.55 0.70 0.62 0.75 0.71 1.12 **0.82**	normo-glycemic normo-insulinemic
	obese	589.4 606.5 635.1 609.6 687.5 669.8 727.3 698.9 **653.0**	40 40 40 40 40 40 40 40	5.11 5.84 5.97 6.25 5.80 6.49 5.62 5.33 **5.80**	3.25 3.44 2.94 2.61 3.50 2.57 3.75 3.29 **3.17**	normo-glycemic hyper-insulinemic
ZDF	lean	376.2 327.8 385.1 395.6 342.0 328.3 401.7 340.5 **362.1**	16 16 16 16 16 16 16 16	6.09 5.79 5.23 5.12 5.53 5.97 6.16 5.38 **5.65**	1.44 1.14 1.99 0.79 0.85 0.57 1.03 1.30 **1.14**	normo-glycemic mildly hyper-insulinemic
	obese	355.6 397.2 337.1 438.0 463.8 451.2 425.9 474.4 **417.9**	16 16 16 16 16 16 16 16	20.41 26.87 19.40 24.23 17.94 25.18 21.07 23.82 **22.37**	1.56 2.98 2.41 3.07 1.84 1.22 2.19 2.63 **2.24**	hyper-glycemic hyper-insulinemic

**Table 2:** Characteristics of the human probands. HbA1c levels of controls (B, G, E) and type 1 diabetic (T1D; C, D, K) and type 2 diabetic (T2D; F, I) patients of various age, gender (F, female; M, male), body weight, and metabolic state are given.

Proband	Age (Years)	Gender	HbA1c (%)	Body Weight	Metabolic State
G	29	F	4.8	lean	control
B	50	F	5.4	lean	control
E	42	M	5.1	lean	control
D	64	F	5.9	overweight	T1D
I	68	M	6.6	overweight	T2D
F	49	F	7.1	obese	T2D
C	41	F	5.5	obese	T1D
K	51	F	7.5	obese	T1D

**Table 3:** Characteristics of plasma HDL from the human probands. HDLs isolated from plasma samples were assayed for the presence of cholesterol (HDL-Chol.), apo A-I, CD73, CD55, and AChE as described in Materials and Methods. Furthermore, total plasma cholesterol (Total Chol.) was determined. (**a**) Means ± SD of four independent measurements each are given. (**b**) Means ± SD of control probands (G, B, E) and T1D/T2D probands (D, I, F, C, K) (^§^ *p* ≤ 0.05 for T1D/T2D vs. Control) are given.

**(a)**						
**Proband**	**HDL-Chol. (mM)**	**apo A-I (mg/dL)**	**CD73 (μg/mL)**	**CD55 (μg/mL)**	**AChE (μg/dL)**	**Total Chol. (mM)**
G	1.45 ± 0.13	161.27 ± 10.40	1.31 ± 0.29	0.12 ± 0.03	0.57 ± 0.19	4.96 ± 0.13
B	1.35 ± 0.18	147.39 ± 8.06	1.37 ± 0.25	0.09 ± 0.03	0.44 ± 0.16	5.04 ± 0.19
E	1.51 ± 0.20	153.84 ± 11.92	1.46 ± 0.19	0.06 ± 0.02	0.35 ± 0.14	4.84 ± 0.24
D	1.79 ± 0.19	174.77 ± 12.51	1.69 ± 0.21	0.14 ± 0.03	0.66 ± 0.14	5.25 ± 0.18
I	1.21 ± 0.23	137.11 ± 10.08	1.82 ± 0.24	0.11 ± 0.02	0.76 ± 0.13	5.16 ± 0.20
F	1.06 ± 0.11	129.63 ± 9.65	1.94 ± 0.31	0.16 ± 0.04	0.70 ± 0.15	5.29 ± 0.23
C	1.85 ± 0.22	180.70 ± 7.91	1.51 ± 0.29	0.10 ± 0.03	0.50 ± 0.11	4.89 ± 0.15
K	1.59 ± 0.18	183.36 ± 8.87	1.77 ± 0.25	0.18 ± 0.02	0.59 ± 0.13	4.74 ± 0.31
**(b)**						
**Proband**	**HDL-Chol. (mM)**	**apo A-I (mg/dL)**	**CD73 (μg/mL)**	**CD55 (μg/mL)**	**AChE (μg/dL)**	**Total Chol. (mM)**
Control	1.44 ± 0.20	154.17 ± 14.87	1.38 ± 0.26	0.09 ± 0.02	0.45 ± 0.17	4.95 ± 0.13
T1D/T2D	1.50 ± 0.18	161.11 ± 13.95	1.75 ^§^ ± 0.22	0.14 ^§^ ± 0.03	0.63 ± 0.15	5.07 ± 0.20

## Data Availability

The datasets generated and analyzed during the current study are available from the corresponding author (G.A.M.; guenter.mueller@helmholtz-muenchen.de) on reasonable request and will be provided as the original SAW data files together with the appropriate SAW software for data visualization and processing, if required, under consideration of the relevant conditions for licensing of FitMaster^®^, SensMaster^®^ and SequenceMaster^®^.

## Supplementary Materials

Supplementary Materials can be found at https://www.mdpi.com/2227-9059/9/3/277/s1. Materials and Methods: Animal handling. GPLD1 activity assay using micelle-like bAChE complexes and chip-based sensing. Reconstitution of micelle-like complexes without bAChE. Measurement of cholesterol concentration in human plasma. Measurement of HDL concentration in human plasma. Preparation and enrichment of hCD73 from human erythrocytes. Figure S1. Measurement of GPLD1 activity in rat serum using chip-based assay. Figure S2. Three modes of capture of micelle-like bAChE complexes by the chips.
