# Supplementary material for: Interaction of Full-Length Glycosylphosphatidylinositol-Anchored Proteins with Serum Proteins and Their Translocation to Cells In Vitro Depend on the (Pre-)Diabetic State in Rats and Humans
Source: Biomedicines. 2021 Mar 10;9(3):277. doi: 10.3390/biomedicines9030277 (PMC8000876; doi:10.3390/biomedicines9030277)
Supplement: Supplementary file 1 [file biomedicines-09-00277-s001.pdf]

Supplementary Materials

Materials

Collagenase (Worthington, CLS type I, 190-250 units/mg) was provided by Biochrom (Berlin, Germany). Bovine serum albumin (BSA; fraction V, defatted), phenylisopropyladensine (PIA), adenosine deaminase (ADA) and polyethylene glycol (PEG) 6000 were obtained from Sigma-Aldrich Chemie GmbH (Munich, Germany). Anti-hCD73 antibody (mouse monoclonal, prepared against CD73 purified from human placenta with no cross-reactivity against mouse/rat CD73, [for dot blotting 1:200]) was provided by Santa Cruz Biotechnology Inc. (sc-32299). Anti-rCD73 antibody (rabbit polyclonal, affinity-purified, IgG isotype, prepared against recombinant full-size human CD73 with cross-reactivity against rat CD73) was obtained from Genetex/Biozol (Eching, Germany). Antibodies against CD55 (ab231061, rabbit polyclonal, affinity-purified, IgG isotype, prepared against the unconjugated His-tagged recombinant peptide corresponding to rat CD55 aa 254-372 and produced in *E. coli*), alkaline phosphatase (aP) (ab95462, rabbit polyclonal, affinity-purified, IgG isotype, prepared against an unconjugated synthetic peptide corresponding to tissue-nonspecific alkaline phosphatase [TNAP]), AChE (ab34533, goat polyclonal, multi-step purified, IgG isotype, biotinylated, prepared against purified AChE from bovine erythrocytes), apolipoprotein A-I (ab52945) (rabbit monoclonal, prepared against a synthetic peptide corresponding to human apolipoprotein A-I aa 1-100 with no cross-reactivity against mouse/rat apo A-I, [for dot blotting 1:500]) and GPLD1 (ab189191, rabbit polyclonal, immunogen affinity-purified, IgG isotype, prepared against a synthetic peptide corresponding to human GPLD1 aa 527-576) were delivered by Abcam (Oxford, UK). Annexin-V (human, recombinant) was purchased from ProSpec (East Brunswick, NJ, USA). 1-ethyl-3-[3-dimethylaminopropyl]carbodiimide (EDC) and N-hydroxysulfosuccinimide (Sulfo-NHS, premium grade) were bought from Pierce/Thermo Scientific (Rockford, IL, USA). Egg york L-α-phosphatidylcholine (PC), bovine brain L-α-phosphatidylserine (PS), bovine brain L-α-lysophosphatidylcholine (lysoPC) and cholesterol (all stored in chloroform) were obtained from Avanti Polar Lipids (Alabaster, AL, USA). Protein A, protein A- and protein G-Sepharose (Cl-4B) and Pha (1,10-phenanthroline) (highest purity available) were from Calbiochem/Merck (Darmstadt, Germany). Proteinase K, PMSF and protease inhibitor cocktail were delivered by Roche Biochemicals (Mannheim, Germany). Polystyrene Bio-Beads SM-2 (20-50 mesh) were bought from Bio-Rad Laboratories (Munich, Germany). NSB Reducer was obtained from GE Healthcare. Rat sera including characterization of the donor rats were made available by Sanofi Pharma Deutschland GmbH (Frankfurt am Main, Germany). Other materials were obtained as described previously [28-30].

Methods

Animal Handling

Male Wistar rats (Crl:WI(WU)) were obtained from Charles River (Sulzfeld, Germany). Rats were housed two *per* cage in an environmentally controlled room with a 12:12-h light–dark circle (light on at 06:00) and *ad libitum* access to water and standard rat chow (17.7 kJ/g, Ssniff diet R/M-H, V1535 with 18% crude protein, 4.7% sugar, and 3.5% crude fat) (Ssniff, Soest, Germany). Serum was collected as described previously [28].

GPLD1 Activity Assay Using Micelle-Like Bovine Acetylcholinesterase (bAChE) Complexes and Chip-Based Sensing

Micelle-like complexes consisting of PC, PS, lysoPC, cholesterol and bAChE, which had been purified from bovine erythrocyte membranes by affinity chromatography using a Sepharose 4B-L-tyrosine-tacrine affinity resin column [S1], as the GPLD1 substrate were prepared by mixing of these constituents at the "optimized" ratio in the detergent-solubilized state and subsequent removal of the detergent by adsorption onto polystyrene Bio-Beads as described previously [30]. This causes the spontaneous assembly of lysoPC together with bAChE and minor amounts of PC, PS and cholesterol into micelle-like complexes, which upon centrifugation were recovered from the supernatant fraction. The "optimized" constituent ratio, lysoPC:PC:cholesterol:bAChE, was 1000:25:25:1 (molar ratio). For incubation with serum samples, 2-25 μL of micelle-like bAChE complexes displaying identical AChE enzymic activity were adjusted to 50 μL with 200 mM Tris/Maleate (pH 7.0), 150 mM NaCl. Sera were diluted 20-fold with 10 mM Tris/HCl (pH 7.4), 150 mM NaCl to reduce the impact of potential inhibitors [100] and then incubated (37 °C, 30 min) with 50 μL of

substrate solution in a total volume of 150 μL of 200 mM Tris/Maleate (pH 7.0), 150 mM $NaCl_2$, 1 mM $MgCl_2$, 0.01 mM zinc acetate, 40 μM $CaCl_2$. The reaction was terminated by the addition of 800 μL of ice-cold 10 mM Hepes/NaOH (pH 7.0), 150 mM NaCl, 0.25 mM Pha and placing on ice. Subsequently, 50-μL portions were subjected to TX-114 partitioning performed as described previously [S2] with modifications [29]. 120-μL portions of the aqueous phase containing the lipolytically cleaved hydrophilic protein moiety of bAChE were injected into α-toxin-coated or "blank" channels of the chip at a flow rate of 36 μL/min. Thereafter, 190 μL of 30 μM PIG41 in 10 mM Tris/HCl (pH 7.4), 150 mM NaCl were injected at a flow rate of 120 μL/min. Phase shift is given (as °) upon correction for a "blank" channel (unspecific interaction of sample components), "buffer alone" (altered viscosity of the serum matrix *vs*. buffer) and a "blank" incubation (amphiphilic-to-hydrophilic conversion of bAChE in the absence of serum) and normalization (set at 0 for 0 sec).

Reconstitution of Micelle-Like Complexes without bAChE

Micelle-like complexes consisting of lysoPC, cholesterol and PC (molar ratio 20:2:1) were prepared by mixing of these constituents in the detergent-solubilized state and subsequent removal of the detergent by adsorption onto polystyrene Bio-Beads SM-2 according to previously published protocols [S3-S6] with the following modifications (see below). This caused the spontaneous assembly of lysoPC and the minor amounts of PC and cholesterol into micelle-like complexes, which upon centrifugation were recovered from the supernatant fractions.

Preparation of Bio-Beads SM-2: 25 g of dry beads were added to 175 mL methanol in a plastic bottle. After rotation of the bottle on a tube rotator (20 rpm, 15 min), the slurry of beads and methanol was poured into a sintered glass funnel on a vacuum flask and the methanol removed by suction. Subsequently, the beads were washed sequentially with another 425 mL of methanol and 2500 mL of deionized water while still residing in the funnel, thereafter suspended in 50 mL of 20 mM Hepes/NaOH (pH 7.5), 100 mM NaCl, 0.02% NaN_3 (HSA) and finally degassed in a vacuum flask (2 h). After having removed any floating beads, the washed beads were stored as a suspension in HSA (20 °C, for up to several months). Just before addition to the reconstitution mixture, an aliquot of beads was weighed out by dispension of slurried beads into a plastic weighing dish, aspiration of any excess liquid from the beads, transfer of the damp beads to a second weighing dish on a balance using a pipet tip or spatula until the weight of the damp beads equaled the desired amount (typically 50 or 350 mg damp weight) and final transfer of the weighed damp beads to the reconstitution mixture (carefully avoiding drying out of the beads before using them).

Reconstitution: LysoPC, PC and cholesterol in chloroform (10 mg/mL each) together with an invariant amout of PS (0.26 μmol) were dispensed into a glass test tube in a total volume of 1 mL at the indicated constituent ratios. After evaporation of the chloroform under a stream of N_2 under atmospheric pressure and then in a speed vac under high vacuum (60 min), the dried phospholipids (lipid films) were dispersed in 250 μL HSA and then completely dissolved by gentle vortexing and incubation (20 °C, 30 min). The hydrated lipid dispersion was exposed to six freezing-thawing cycles (-180 °C/+25 °C) and then passed 40 times through a polycarbonate membrane (0.2 μm) with a mini-extruder (Avanti Polar Lipids). Reconstitution was initiated by the addition of 50 mg damp Bio-Beads SM-2 to the tube and rotation on a tube rotator (20 rpm, 90 min, 20 °C). After addition of another 350 mg (damp weight) of Bio-Beads SM-2 and rotation (180 min), the beads were allowed to settle (5 min). For separation and recovery, 200-μL portions of the supernatant were centrifuged (400,000x*g*, 1 h, 4 °C; Beckman TL-100 ultracentrifuge, TLA-100 rotor, 95,000 rpm). The supernatants containing the micelle-like complexes were subjected to a second cycle of detergent absorption with Bio-Beads SM-2 (50 and 350 mg, see above). The final clear supernatants were subsequently purified by discontinuous flotation gradients [S3-S6]. For this, 3-mL portions were thoroughly mixed with 3 mL of sucrose (60% *w*/*v*) in 20 mM Pipes/KOH (pH 7.2), 100 mM K_2SO_4 at the bottom of 10-mL centrifuge tubes. 1-mL cushions of 10% and 2.5% sucrose (*w*/*v*) were successively layered above, followed by 2 mL of buffer. After centrifugation of the density gradients (30,000 rpm, 2 h, 4 °C, Beckman SW41 rotor), 0.5-mL fractions were removed from the 2.5%-sucrose buffer interface resulting in about 5- to 6-fold concentration of the purified micelle-like complexes lacking (bAChE) protein.

Measurement of Total Cholesterol Concentration in Human Plasma

Cholesterol was determined by an enzymatic method [S7] using a commercially available kit (CHOD-PAP, Roche Biochemicals, Mannheim, FRG) or, alternatively, by a two-step dextran sulfate magnesium precipitation method [S8].

Measurement of HDL Concentration in Human Plasma

Gradient fractions obtained by serial ultracentrifuation of plasma lipoproteins for the isolation of HDL were assayed for cholesterol. Cholesterol-containing fractions were subjected to purification by immune adsorbent/affinity chromatography for the enrichment of HDL harbouring apo A-I, CD55 and CD73 with the use of anti-apo A-I and anti-CD55 antibodies and 5'-AMP-Sepharose 4B beads, respectively, in a batch procedure. The anti-apo A-I and CD55 immune adsorbents were prepared by covalent coupling of anti-apo A-I and CD55 antibodies to CNBr-activated Sepharose 4B at a ratio of 5-8 and 1-2 mg/mL, respectively, of gel according to the protocol of the manufacturer (LKB/Pharmacia) and published procedures [S9,S10]. In brief, the antibody-Sepharose gels were extensively washed with several cycles of $NaHCO_3$/NaCl and NaAc/NaCl and then with 0.1 M acetic acid (pH 3.0), 0.1 M glycine (pH 2.7). Finally, the gels were re-equilibrated with Tris/HCl containing 0.02% Merthiolate and stored (4 °C). For immune adsorption, 2 mg of anti-apo A-I and CD55 antibodies or 5'-AMP Sepharose 4B (LKB/Pharmacia Fine Chemicals, Freiburg, Germany) in 200 μL of 20 mM Tris/HCl (pH 7.6), 0.5 M NaCl, 0.1% TX-100 were mixed with 50 μL of sample in 1-mL Epppendorf cups and then incubated (16 h, 4 °C, 30 head-to-bottom rotations *per* min). Lipoproteins that did not bind to the affinity materials were washed off with 20 mM Tris/NaCl (pH 7.6), 1 mM EDTA and then concentrated under vacuum to a volume between 0.75 and 1.25 mL in a Micro-Confilt concentrator (Biomolecular Dynamics Inc.) using protein dialysis membrane (15 kDa cut-off, Biomolecular Dynamics). Non-specifically bound lipoproteins were removed by washing the gel with $NaHCO_3$/NaCl and NaAc/NaCl, 1 mM EDTA. Elution of lipoproteins from the affinity materials was provoked by addition of 750 μL of 5 mM AMP, 20 mM Tris/HCl (pH 7.6), 0.1% TX-100 (for CD73-harbouring HDL) or 750 μL of 3 M sodium thiocyanate, 20 mM sodium phosphate buffer (pH 7.0) (for apo A-I- and CD55-harbouring HDL). After centrifugation (10,000x*g*, 5 min, 4 °C), the supernatants containing HDL which harbour CD73, apo A-I or CD55, respectively, were rescued and immediately filtered through a column packed with Sephadex G-25 (LKB/Pharmacia, Freiburg, Germany) and then dialyzed against Tris/HCl (pH 7.4), 150 mM NaCl, 1 mM EDTA for removal of AMP and thiocyanate. Following concentration under vacuum to volumes to one third of the starting plasma volume using a Micro-Confilt concentrator, the relative amounts of CD73, apo A-I and CD55 were determined by dot blotting with corresponding antibodies.

Preparation and Enrichment of hCD73 (5′-Nucleotidase) from Human Erythrocytes

CD73 was purified according to published procedures [S11,S12] with the following modifications: After centrifugation of acid-citrate-dextrose blood (outdated bank blood) and aspiration of the plasma and buffy layer, the packed cells were resuspended in PBS and then filtered through a column with a 5-cm bed prepared by mixing equal amounts of microcrystalline cellulose and α-cellulose for the removal of leukocytes and platelets. Thereafter, the erythrocytes were washed with PBS through the cellulose, then washed three times with PBS, suspended in an equal volume of PBS and finally hemolysed and extracted by addition of five volumes of 10 mM Tris/HCl (pH 7.6) containing 0.1% BATC. The mixture was passed through a DEAE-cellulose column (sintered glass, 10 cm length, 5 cm bed, equilibrated with 10 mM Tris/HCl, pH 7.6, 0.05% BATC). After washing of the column till complete elution of all hemoglobin with 10 mM Tris/HCl, 0.05% BATC under gentle suction, CD73 was eluted by washing with 500 mL of 10 mM Tris/HCl (pH 7.6), 0.05% BATC, 175 mM NaCl. Further enrichment of CD73 was achieved by rate-zonal separation. For this, 21.5 mL of the erythrocyte hemolysate/extract were injected into the central opening of an MSE BXIV Ti rotor harbouring a linear gradient of 100 mL, each, of 10% and 30% sucrose, 10 mM Tris/HCl (pH 7.6), 0.05% BATC (MSE automatic gradient former). After filling of the rotor from the outer edge with 112.5 mL of 30% sucrose and subsequent displacement from the inner edge by the erythrocyte hemolysate/extract, the gradient was overlayed by 95 mL of 10 mM Tris/HCl (pH 7.6), 0.05% BATC. After centrifugation (43,000 rpm, 24 h, MSE 65 centrifuge), 60% sucrose was pumped into the outer edge of the rotor to unload the contents of the rotor. 12.5-mL fractions were collected manually and then assayed for 5'-nucleotidase activity [S13,S14]. Fractions exhibiting 5'-nucleotidase activity were pooled (35-50 mL) and then concentrated to 3-4 mL by pressure ultrafiltration (Amicon cell, XM-50 filters). Final enrichment was

performed by gel filtration (Sepharose 6B, equilibrated with 10 mM Tris/HCl, pH 7.6, 0.05% BATC). Peaks of 5'-nucleotidase activity were concentrated by pressure ultrafiltration. The enriched erythrocyte hCD73 was stored at -80 °C.

Since the majority of the enriched erythrocyte CD73 fractions were contained detectable non-specific phosphatase activity, the 5'-nucleotidase activity was measured by coupling the reaction to adenosine deaminase (ADA) (in excess) activity and monitoring the conversion of 5'-AMP *via* adenosine to inosine [S13,S14]. The assay mixture in a 1-cm light-path cuvette harboured 10-100 µL sample, 0.08 µmol of AMP, 0.09 units of ADA (type 4, Sigma, Deisenhofen, Germany) and 43 µmol of Tris/HCl (pH 7.6) in a total volume of 0.5 mL. The reaction was initiated by the addition of sample and run for 30 min at 20 °C (water-jacketed cuvette). The absorbance change at 265 µm was measured (Hitachi 200-10 spectrophotometer). The absorbance change *per* minute was converted to µmol of AMP hydrolysed *per* minute by the following relationship: µmol/min = absorbance change *per* minute through 8.1 (derived from the difference in the extinction coefficients of adenosine and inosine at 265 µm. In case of absence of detectable non-specific phosphatase activity in the CD73 fractions, 5'-nucleotidase activity was assayed by measurement of orthophosphate released. For this, the reaction mixture contained 5 mM 5'-AMP, 33 mM Tris/HCl (pH 7.6) and sample in a total volume of 0.5 mL. The reaction was started by addition of the sample and terminated by addition of 10% trichloroacetic acid. Orthophosphate liberated in the reaction mixture was determined by the method of Fiske-Subbarow [S15] with a slight modification, in which the deep color of phosphomolybdate was developed in the presence of TX-100 at a final concentration of 0.35%.

Measurement of the specific 5'-nucleotidase activity (which was performed in triplicate with each sample) in the unlysed washed erythrocytes, erythrocyte hemolysate/extract, peak fractions from the rate-zonal separation and peak fractions from the gel filtration revealed a final enrichment of 125- to up to 140-fold with a yield of 14-20%. SDS-polyacrylamide gel electrophoresis (4-20% polyacrylamide, tricine buffer) and subsequent Coomassie Blue staining led to the detection of three major bands and six to eight minor ones. One of the major bands, which accounted for about half of the total protein, corresponded to 71 kDa and was identified as CD73 by subsequent immune blotting with anti-hCD73 antibody.

Supplementary Figures

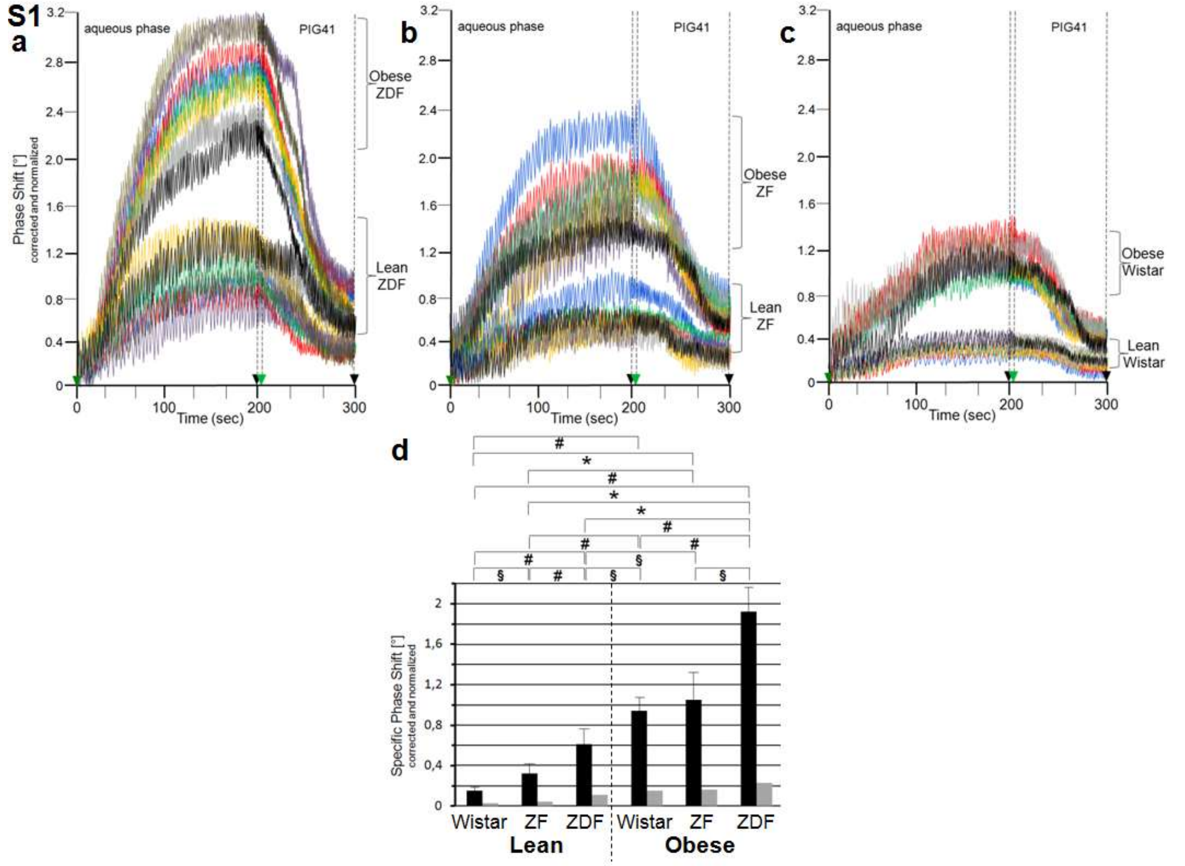

Figure S1. Measurement of GPLD1 activity in rat serum using chip-based assay. (**a-c**) bAChE reconstituted into micelle-like complexes at the "optimized" constituent ratio was incubated with identical volumes of serum from the six rat groups. After TX-114 partitioning of the total incubation mixtures, the aqueous phase harbouring the cleaved protein moiety of bAChE was injected into α-toxin-coated chips (time point 0). bAChE specifically captured *via* the retained GPI core glycan was released by injection of PIG41 (30 μM) at time point 203 sec. Measured phase shift was corrected for unspecific capture of serum components (uncoated control channel) and altered viscosity (*vs*. buffer) of the sample fluids and normalized for the varying responsiveness of distinct chips towards GPI-AP capture. (**a-c**) Measurements of sera from eight rats *per* group (performed with the same chip each and repeated two times with different preparations of micelle-like bAChE complexes and yielding similar results) are shown (12 regeneration cycles *per* chip; representatives only). (**d**) Specific phase shift was calculated as the difference between the total phase shift upon injection of the aqueous phase (start of PIG41 injection at time point 203 sec) and the unspecific phase shift remaining left upon termination of PIG41 injection (time point 300 sec) and are given as means ± SD for the absence (n = 8; measured in triplicate) (black bars) and presence (n = 1, pooled samples, single measurement) (grey bars) of Pha (0.5 mM). It represents a direct measure for GPLD1 activity as was confirmed by almost complete abrogation by Pha due to the Ca^{2+} dependence of GPLD1. The comparison of the specific phase shift between the rat groups is given for the absence of Pha, only (black bars) (* $p \leq 0.01$, # $p \leq 0.02$, § $p \leq 0.05$).

Results: For determination of GLPD1 activity, the conversion of amphiphilic to hydrophilic dimeric bovine erythrocyte AChE (bAChE), which represents a typical GPI-AP [S13,S14], upon GPI anchor cleavage [S16,S17] was assessed by TX-114 partitioning, which leads to enrichment of the amphiphilic full-length and hydrophilic cleaved versions of bAChE in the detergent-enriched and aqueous phases, respectively. The amount of hydrophilic bAChE having retained the GPI core glycan as determined by capture through α-toxin-coated channels represents a measure for lipolytic, rather than proteolytic, removal of the GPI anchor. On the basis of missing evidence for the expression of a soluble GPI-specific phospholipase C in mammalian serum [1,5,6,36,37,S16,S17], it is reasonable to assume that the chip-based assay is specific for GPLD1.

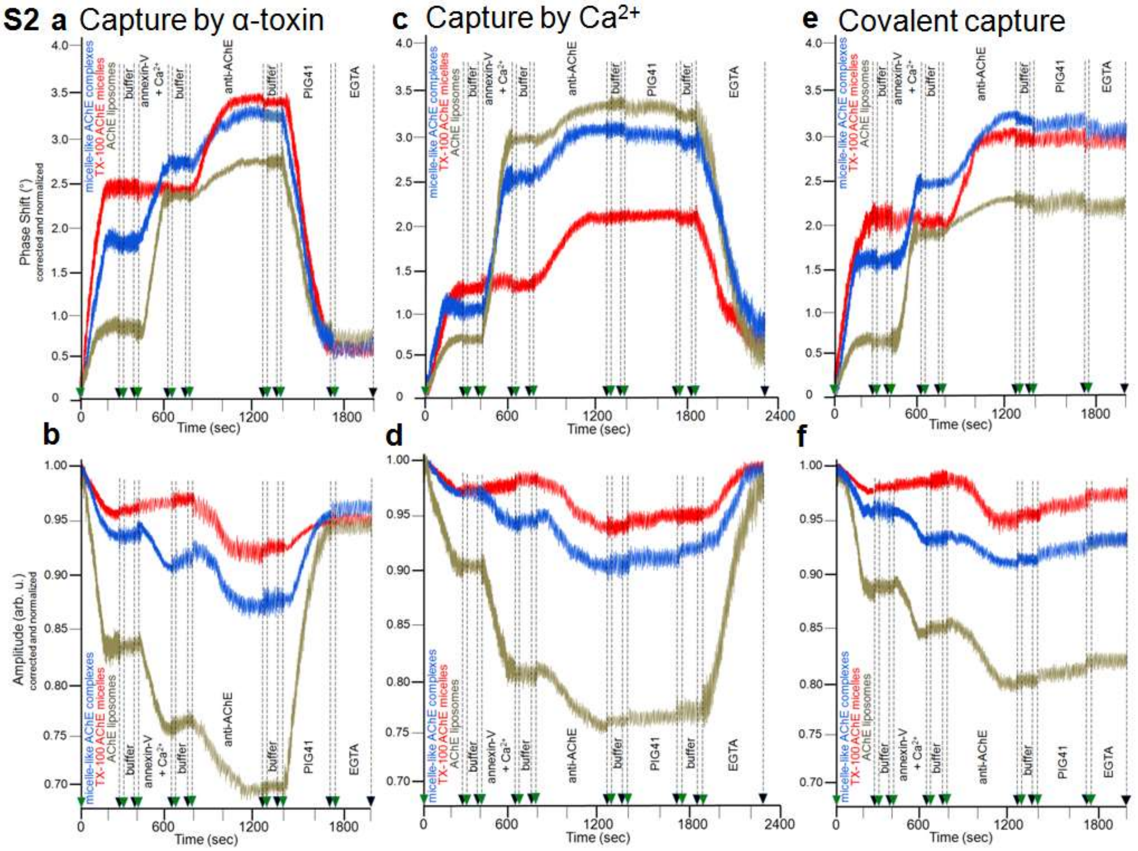

Figure S2. Three modes of capture of micelle-like bAChE complexes by the chips. bAChE reconstituted into micelle-like complexes at the "optimized" constituent ratio (blue curves), TX-100-lysoPC micelles (red curves) or liposomes (brown curves) was injected into α-toxin-coated chips (**a**,**b**), into uncoated chips in the presence of Ca^{2+} (**c**,**d**) or into EDC/NHS-activated chips for covalent capture (**e**,**f**). Following washing, the presence of phospholipids and bAChE in the chip-captured structures was detected by sequential injection of annexin-V together with Ca^{2+} and anti-AChE antibody with additional washing steps between and at the end of the injections. The nature of the interaction of bAChE with the chip surface was demonstrated by successive injection of PIG41 (10 µM) and EGTA (5 mM). The measured phase shift and amplitude (set at 0 and 1 arb. unit, respectively, for time point 0) were corrected for unspecific interaction (uncoated control channel) and altered viscosity of the sample fluid (*vs*. buffer) and normalized for the varying responsiveness of distinct chips towards capture of GPI-APs by α-toxin (**a**,**b**), by Ca^{2+} (**c**,**d**) and by covalent means (**e**,**f**). The experiment was repeated once using distinct chips and different preparations of complexes, micelles and liposomes with similar results (representatives shown).

Results: (**a**,**c**,**e**) The efficacy of capture of micelle-like bAChE complexes was compared with that of bAChE liposomes and TX-100 bAChE micelles (containing minute amounts of [lyso]phosphatidylcholine) by sequential injection of annexin-V and anti-AChE antibody and found to vary only by maximal 50% between the three modes of capture on basis of identical amounts of bAChE reconstituted into the complexes, liposomes and micelles and measurement of the increases in phase shift as parameter for the amount of chip-captured materials. (**b**,**d**,**f**) The physical characteristics of the complexes, liposomes and micelles remained largely unaffected by the mode of capture as revealed by the similar reductions in amplitude as parameter for the viscoelasticity of the chip-captured materials. On the basis of similar amounts captured by the chips (as indicated by phase shift), the decreases in amplitude upon injection of annexin-V and anti-AChE antibody were found to be most pronounced for bAChE liposomes. This argued for higher viscosity of liposomes in comparison to micelle-like bAChE complexes and TX-100 bAChE micelles, which both exhibit a more rigid structure and thereby higher elasticity. The mode of capture of the complexes, liposomes and micelles by chips *via* α-toxin and Ca^{2+} was confirmed by consecutive injection of excess of PIG41 (**a**,**b**) and

EGTA (**c**,**d**), respectively, which led to complete loss of phase shift increase and amplitude reduction, but did not affect covalent capture (**e**,**f**).

References

S1. Kaya, H.B.; Özcan, B.; Sisecioglu, M.; Ozdemir, H. Purification of acetylcholinesterase by 9-amino-1,2,3,4-tetrahydroacridine from human erthrocytes. *Appl. Biochem. Biotechnol.* **2013**, *170*, 198-209.
S2. Bordier, C. Phase separation of integral membrane proteins in Triton X-114 solutions. *J. Biol. Chem.* **1981**, *256*, 1604-1607.
S3. Agrand, M.; Briolay, A.; Ronzon, F.; Roux, B. Detergent-mediated reconstitution of a glycosyl-phosphatidylinositol-protein into liposomes. *Eur. J. Biochem.* **1997**, *250*, 168-176.
S4. Morandat, S.; Bortolato, M.; Roux, B. Cholesterol-dependent insertion of glycosylphosphatidylinositol-anchored enzyme. *Biochim. Biophys. Acta* **2002**, *1564*, 473-478.
S5. Rigaud, J.-L.; Paternostre, M.-T.; Bluzat, A. Mechanisms of membrane protein insertion into liposomes during reconstitution procedures involving the use of detergents. 2 Incorporation of the light-driven proton pump bacteriorhodopsin. *Biochemistry* **1988**, *27*, 267-2688.
S6. Smith, S.A.; Morrissey, J.H. Rapid and efficient incorporation of tissue factor into liposomes. *J. Thromb. Haemost.* **2004**, *2*, 1155-1162.
S7. Röschlau, P.; Bernt, E.; Gruber, W. Enzymatische Bestimmung des Gesamtcholesterins in Serum. *Z. Klin. Biochem*. **1974**, *12*, 226-232.
S8. Warnick, G.R.; Benderson, J.M.; Albers, J.J. *Clin. Chem*. **1982**, *1928*, 28, 1574A.
S9. Cheung, M.C.; Albers, J.J. Characterization of lipoprotein particles isolated by immunoaffinity chromatography. Particles containing A-I and A-II and particles containing A-I but no A-II. *J. Biol. Chem*. **1984**, *259*, 12201-12209.
S10. Cheung, M.C.; Wolf, A.C. In vitro transformations´of apo-A-I-containing lipoprotein subpopulations: role of lecithin:, cholesterol acyltransferase and apoB-containing lipoproteins. *J. Lipid Res*. **1989**, *30*, 499-509.
S11. Torrance, J.D.; Whittaker, D.; Beutler, E. Purification and properties of human erythrocyte pyrimidine 5′-nucleotidase. *Proc. Natl. Acad. USA* **1977**, *74*, 3701-3704.
S12. Yamamoto, H.; Tomioka, K.; Kawai, H.; Endo, K. Purification and properties of %′-nucleotidase from Cod muscle. *Agric. Biol. Chem.* **1986**, *50*, 1123-1129.
S13. Rosenberry, T.L.; Roberts, W.L.; Haas, R. Glycolipid membrane-binding domain of human erythrocyte acetylcholinesterase. *Fed. Proc*. **1986**, *45*, 2970-2975.
S14. Taguchi, R.; Ikezawa, H. Properties of bovine erythrocyte acetylcholinesterase solubilized by phosphatidylinositol-specific phospholipase C1. *J. Biochem*. **1987**, *102*, 803-811.
S15. Fiske, C.H.; Subbarow, Y. The colorimetric determination of phosphorus. *J. Biol. Chem.* **1925**, *66*, 375-400.
S16. Müller, G.; Dearey, E.-A.; Korndörfer, A.; Bandlow, W. Stimulation of a glycosylphosphatidylinositol-specific phospholipase by insulin and the sulfonylurea, glimepiride, in rat adipocytes depends on increased glucose transport. *J. Cell Biol.* **1994**, *126*, 761-780.
S17.Müller, G.; Schulz, A.; Wied, S.; Frick, W. Regulation of lipid raft proteins by glimepiride- and insulin-induced glycosylphosphatidylinositol-specific phospholipase C in rat adipocytes. *Biochem. Pharmacol.* **2005**, *69*, 761-780.
